# Basis for a neuronal version of Grover's quantum algorithm

**DOI:** 10.3389/fnmol.2014.00029

**Published:** 2014-04-17

**Authors:** Kevin B. Clark

**Affiliations:** ^1^Research and Development Service, Veterans Affairs Greater Los Angeles Healthcare SystemLos Angeles, CA, USA; ^2^Complex Biological Systems AllianceNorth Andover, MA, USA

**Keywords:** biotechnology, calcium-induced calcium reactions (CICRs), cellular decision making, classical and quantum computation, inositol 1,4,5-trisphosphate receptors (IP_3_Rs), intracellular calcium, neuronal plasticity, quantum molecular networks and memory

## Abstract

Grover's quantum (search) algorithm exploits principles of quantum information theory and computation to surpass the strong Church–Turing limit governing classical computers. The algorithm initializes a search field into superposed *N* (eigen)states to later execute nonclassical “subroutines” involving unitary phase shifts of measured states and to produce root-rate or quadratic gain in the algorithmic time (*O*(*N*^1/2^)) needed to find some “target” solution *m*. Akin to this fast technological search algorithm, single eukaryotic cells, such as differentiated neurons, perform natural quadratic speed-up in the search for appropriate store-operated Ca^2+^ response regulation of, among other processes, protein and lipid biosynthesis, cell energetics, stress responses, cell fate and death, synaptic plasticity, and immunoprotection. Such speed-up in cellular decision making results from spatiotemporal dynamics of networked intracellular Ca^2+^-induced Ca^2+^ release and the search (or signaling) velocity of Ca^2+^ wave propagation. As chemical processes, such as the duration of Ca^2+^ mobilization, become rate-limiting over interstore distances, Ca^2+^ waves quadratically decrease interstore-travel time from slow saltatory to fast continuous gradients proportional to the square-root of the classical Ca^2+^ diffusion coefficient, *D*^1/2^, matching the computing efficiency of Grover's quantum algorithm. In this Hypothesis and Theory article, I elaborate on these traits using a fire-diffuse-fire model of store-operated cytosolic Ca^2+^ signaling valid for glutamatergic neurons. Salient model features corresponding to Grover's quantum algorithm are parameterized to meet requirements for the Oracle Hadamard transform and Grover's iteration. A neuronal version of Grover's quantum algorithm figures to benefit signal coincidence detection and integration, bidirectional synaptic plasticity, and other vital cell functions by rapidly selecting, ordering, and/or counting optional response regulation choices.

## Introduction

Modern analog, digital, and quantum descriptions of phylogenetically diverse cell functions (e.g., Monod and Jacob, 1961; McAdams and Shapiro, 1995; McAdams and Arkin, 2000) date to Twentieth-century revelations in computational and information sciences (e.g., Szilárd, 1929; Turing, 1936; Shannon, 1938, 1948a,b; Landauer, 1961; Feynman, 1982; Deutsch, 1985). Continued advances in systems biology, synthetic biology, and micro- and nanobiotechnology increasingly drive states-of-knowledge and -art in computational cell biology toward trends in logic gate, circuit, and algorithm designs (e.g., Ehrenfeucht et al., 2003; Amos, 2006; Baumgardner et al., 2009; Friedland et al., 2009; Adamatzky, 2010; Clark, 2010a,b,c,d, 2011, 2012b, 2013a; Norris et al., 2011; Karafyllidis, 2012; Mehta and Schwab, 2012; Daniel et al., 2013; Goñi-Moreno et al., 2013; Ji et al., 2013), especially for “programmable” group and solitary cellular decisions mediated by genetic, epigenetic, and somatic regulatory networks. Unsurprisingly, given their preeminent status as computational units (cf. Koch and Segev, 2000; Grillner, 2006), single neurons are still favored models for bioinspired smart technologies (e.g., Liu et al., 2013). Yet, despite technological interests in neuronal information processing attributes, serious application of quantum computational approaches toward study of adaptive cybernetic-like neuron behavior and physiology remains disappointingly slow, except as it may broadly relate to more-or-less controversial debates over the statistical mechanics nature of consciousness, decision making, and other psychological states and functions of humans and animals (cf. Beck and Eccles, 1992; Hameroff, 1994, 2012; Tegmark, 2000; Schwartz et al., 2005; Khrennikov, 2009; Pothos and Busemeyer, 2013a,b). The strange properties of quantum mechanics, such as superposition, entanglement, interference, and tunneling (Box 1), can be harnessed to enhance the information storage capacity, processing speed, and fault tolerance of man-made computational systems (cf. Nielsen and Chuang, 2000). Over recent decades, quantum information theorists have steadily identified and adapted quantum computational constructs believed to outperform the classical universal Turing machine (Feynman, 1982; Deutsch, 1985), supported by the strong Church–Turing thesis to be the upper limit for powerful computational devices obeying classical information theory and physicochemical laws. Actual physical quantum computers are only now moving out of proof-of-concept stage due to gradual progress in innovating suitable, if not optimal, device architectures, such as optical lattices, ion traps, nuclear magnetic resonators, quantum dots, and other technologies (e.g., Vandersypen et al., 2001; DiCarlo et al., 2009; Politi et al., 2009; Johnson et al., 2011). Nonetheless, algorithms built from quantum gates and circuits offer exciting practical, though often intuitively difficult, ways for obtaining performance characteristics better than those exhibited by classical processors. For example, several general classes of quantum algorithms based on Shor's quantum Fourier transform (Shor, 1994), the Deutsch–Jozsa algorithm, and Grover's quantum algorithm (Grover, 1996) are known. Through a series of quantum logic gates, Shor's quantum Fourier transform enables a fast two-register eigenvalue phase-estimation procedure to be executed on eigenstates of a unitary operator put into quantum superposition. Phase-estimation subroutines, in turn, serve as modules for other algorithms that exponentially decrease the number of operations required to solve important problems, such as related order-finding and hidden-subgroup problems, judged intractable with classical computers. Alternately, Grover's quantum algorithm, also termed Grover's quantum search algorithm, the quantum search algorithm, or the fast search algorithm, reaches only root-rate or quadratic operating improvements when compared to classical algorithms searching or counting elements of unstructured databases. This single-register algorithm initializes the search field containing target solutions into a uniform superposition state via a quantum transformation. A quantum subroutine called the Grover's operator or iteration then conditionally shifts or rotates the phase of certain computational bases until search solutions become found.

Box 1 **Glossary of terminology.**

**Bell Basis States or Einstein–Podolsky–Rosen Pairs**

Named respectively after John Bell and Albert Einstein, Nathan Rosen, and Boris Podolsky, these four orthonormal quantum states, spanning the two-qubit state space, form an essential computational basis for many fundamentally useful applications of quantum information theory. The states or pairs are: |ψ〉_00_ = (|00〉 + |11〉)/2^1/2^, |ψ〉_01_ = (|01〉 + |10〉)/2^1/2^, |ψ〉_10_ = (|00〉 − |11〉)/2^1/2^, |ψ〉_11_ = (|01〉 − |10〉)/2^1/2^. Quantum entanglement between the two qubits of a state permits secure cryptographic protocols, such as quantum teleportation, and compressed information encoding and storage, such as superdense coding and quantum memory.

**Church–Turing Limit**

Upper computational bound of efficiency for classical computers independently determined by Alonzo Church and Alan Turing. The Church–Turing limit emerges from the Church–Turing thesis or conjecture, which equates functions computable on a Turing machine with those computable by an algorithm. The strong limit is believed to subtend allowable complexity of computations performed by quantum computers, as captured in David Deutsch's rigorous conceptualization of universal quantum computers.

**Eigenstates, Eigenvalues, and Eigenspaces**

An eigenstate or eigenvector, |*v*〉, is a nonzero state in a state space operated on by a linear function *L*, so that *L*|*v*〉 = ω|*v*〉 with complex eigenvalue ω. Eigenstates and eigenvalues are ascertained from the characteristic function, *c*(λ) = det|*L* − λ*I*|, where det is the determinant function. The eigenspace of ω, a subset of the state space on which *L* acts, is the set of eigenstates with the eigenvalue ω.

**Entropic Uncertainty Principle**

Information or entropy principle first derived by Iwo Bialynicki-Birula and Jerzy Mycielski and by David Deutsch from Werner Heisenberg's uncertainty principle. The principle is formally expressed in the strong condition as *H*(*Q*) + *H*(*R*) ≥ 2 log_2_[1/*f*(*Q, R*)], where *H*(*Q*) and *H*(*R*) are the Shannon entropies of respective spectrally decomposed measurements *Q* and *R* of quantum state |ψ〉 with probability distributions *p*(*q*) and *p*(*r*) and maximum fidelity or inner product *f*(*Q, R*) = max_*q,r*_|〈*q*|*r*〉| between eigenvectors |*q*〉 and |*r*〉. As with Heisenberg's version involving the standard deviation of observables, the entropic uncertainty principle places an upper bound on attainable knowledge about quantum systems.

**Hadamard Transformation**

An important step for quantum parallel computing, the Hadamard transformation applies the Hadamard gate *n* times to *n* input qubits to initialize the data register of a quantum information system into superposition, so that multiple values of an index integer *x* can be simultaneously analyzed by a single function *f*. Common notation for the Hadamard transform is H^⊗*n*^.

**Hermitian Operator**

A linear operator, also known as a self-adjoint operator, imposed on a vector space *V*. For a Hermitian operator *L*, there is a unique linear operator *L*^†^ acting on *V*, so that an adjoint or conjugate vector |*v*〉^†^ exists for every |*v*〉 in *V*. That is, *L* = *L*^†^, |*v*〉 = |*v*〉^†^, and *LL*^†^ = *L*^†^*L* when *L* is normal.

**Landauer's Principle**

Principle postulated by Rolf Landauer to define the relationship between energy and computation. Landauer improved earlier ideas of Leó Szilárd, John von Neumann, and other theorists to concretize the minimum amount of energy/information consumed during irreversible operations. Although Landauer's principle applies generally to energy/information dissipated as heat from work, it is usually placed into the context of memory erasure. For example, for a biological or technological computer with exhausted finite memory capacity, it is necessary to erase information for further computations. Landauer's principle states at least *k*_B_ *T*ln2 of energy, where *k*_B_ is Boltzmann's constant and *T* is ambient temperature in degrees Kelvin, must be transferred to the environment for erasure of one bit of information. The corresponding entropy rendering of Landauer's principle may be written without variable *T*.

**Quantum Gates and Circuits**

Logic gates and circuits constructed to perform operations based on quantum mechanics and information theory. Popular unitary single-qubit gates in matrix form include, for instance, the quantum Identity $I=\left[\begin{array}{c} 1\\ 0 \end{array}\begin{array}{c} 0\\ 1 \end{array}\right]$ gate, the quantum NOT or Pauli $X=\left[\begin{array}{c} 0\\ 1 \end{array}\begin{array}{c} 1\\ 0 \end{array}\right]$, Pauli $Y=\left[\begin{array}{c} 0\\ i \end{array}\begin{array}{c} -i\\ 0 \end{array}\right]$, and quantum Flip or Pauli $Z=\left[\begin{array}{c} 1\\ 0 \end{array}\begin{array}{c} 0\\ -1 \end{array}\right]$ gates, the Hadamard $H=1/2^{1/2}\left[\begin{array}{c} 1\\ 1 \end{array}\begin{array}{c} 1\\ -1 \end{array}\right]$ gate, and the Phase $S=\left[\begin{array}{c} 1\\ 0 \end{array}\begin{array}{c} 0\\ i \end{array}\right]$ gate. These and other gates may be assembled into quantum circuits, such as multiple-qubit controlled-NOT, controlled-Phase, controlled-Swap or Fredkin, and Toffoli gates, also used to transform input qubits. The above single-qubit gates establish with other quantum gates a discrete subset of logical primitives (i.e., gates and/or circuits) capable of unitary transformation and of emulating any other transformation to approximate computational universality.

**Quantum Mechanical Properties**

Statistical wave-particle features of quantum mechanical systems, such as quantum superposition, entanglement, interference, and tunneling, not observed for deterministic classical Newtonian physical or Shannon informational systems. Quantum superposition describes the linear combination or addition of state or vector solutions to Schrödinger's wave equation (or other quantum state equation permutations). When superposed states are indistinguishable, they are said to be entangled. Quantum interference is the disruption of state or vector (e.g., a wave or particle) spacetime trajectories. And quantum tunneling is the transition of one state to another without surmounting classical energy barriers required for transformation in classical physics. Such probabilistic effects are useful for development of information technologies and additional purposes.

**Quantum Networks**

Technological and biological networks whose connectivity tends to obey either Bose-Einstein or Fermi-Dirac quantum statistics rather than classical Maxwell-Boltzmann statistics. The strength of each network node is described as a separate fitness or energy level and nodal links take on the identity of particle states functioning under associative-like preferential attachment rules. In such cases, control parameter *T* (i.e., local absolute temperature), which dictates system behavior, is often replaced with a computational annealing parameter, such as space, time, or the “critical tunneling field strength.” Quantum networks may display the network analogs of Bose-Einstein condensation and the Pauli exclusion principle depending on statistical parameters. In addition, rate of state transitions or computational decisions in a quantum network follows nonlinear first-order Arrhenius kinetics associated with quantum tunneling, also making it a computational or network analog of the physical phenomenon. These properties of quantum networks strongly compare with networks capable of certain associative forms of learning and memory, such as Hebbian-type learning dependent upon mutually weighted nodal or synaptic strengths.

**Schrödinger Wave Function**

A solution to Erwin Schrödinger's wave equation used to describe the statistical nature of eigenstates that exhibit wave-particle duality. The wave function gives the probable energy of a wave in a 4D spacetime interval. Each wave is associated with a respective wave number related to wavelength or an energy level known as a quantum. Because the wave equation is additive, the distribution of wave numbers or energy levels occurring over the spacetime interval may be combined into a mixed or superposed wave packet. Pure states are represented by a single wave number or quantum.

**Unitary Operator**

A mathematical operation performed on a state space that satisfies the identity operator by being normal, having a spectral decomposition (i.e., capable of being reduced to additive components), and preserving the inner product of two vectors, such as a unitary phase shift of 180° from state or vector |0〉 to |1〉 or |1〉 to |0〉 residing on the unit circle or the unit 3D sphere—the Bloch sphere.

**Universal Turing Machine**

An idealized computational machine with unbounded memory belonging to a class of devices introduced by Alan Turing, the universal Turing machine is one of the most powerful classical computers conceived and serves as model to examine issues of computational complexity. All Turing machines are prototype modern programmable computers capable of executing algorithmic routines of different complexity. They consist of four essential components: (1) a microprocessor-like finite state control that coordinates computing action, (2) a program, (3) a memory tape, and (4) a read-write tape head that points to the tape location currently accessible for read-write operations. Unlike other Turing machines, the only variable component maintained by the universal Turing machine is the initial contents of the tape. Such a configuration is deceivably powerful and enables the universal Turing machine to emulate or simulate the processing of all other Turing machines, even more powerful ones.

Similar processing advantages arising from quantum mechanics may exist for natural computations performed by live biological systems, particularly at the level of single cells and their subcellular components. Mounting evidence from decades of analytical and experimental research continues to oppose the conventional tenet that quantum mechanical phenomena exert, at best, trivial influences over bioprocesses (cf. Davies, 2004). Criticisms still tend to concentrate on the capacity of biological systems to settle or cohere into a quantum regime long enough to accomplish quantum computation (cf. Tegmark, 2000; Davies, 2004; Reimers et al., 2009; Wolynes, 2009; Trevors and Masson, 2010). However, issues regarding quantum decoherence, the collapse of the Schrödinger wave function into a single classical or macroscopic state due to thermodynamic processes involving a system and its environment, are less problematic for cellular enzymatic processes reliant on small, thermally-shielded protein reaction sites and/or on local temperature gradients which can force cellular substrate from decoherent to coherent activity (cf. Davies, 2004). Considering these factors, a number of substrate essential for cellular computations are already associated with quantum performance characteristics, such as cytoskeletal lattices (Hameroff, 1994; Matsuno, 2006; Craddock et al., 2009), photosynthetic protein complexes (Hu et al., 1998; Sener et al., 2005), the citric acid cycle (Matsuno, 2006)and metabolism (Demetrius, 2003), molecular ratchets (Matsuno, 1999, 2006; McFadden and Al-Khalili, 1999; Patel, 2001; Cooper, 2009), molecule folding (Gutin et al., 1996; Cieplak and Hoang, 2003), synaptic boutons and vesicles (Beck and Eccles, 1992; Schwartz et al., 2005), long-range enzymatic activity (Fröhlich, 1968, 2004; see Reimers et al., 2009 for a dissenting view), odorant receptors (Turin, 1996; Brookes et al., 2007; Solov'yov et al., 2012), and second-messenger cascades (Clark, 2010a,b,c,d, 2011, 2012b). Quantum effects at both informational and physical degrees of freedom thus *seem* to appear in every major aspect of cell structure and function, from sensory transduction to gene expression to cellular metabolism to cell motility (Clark, 2011, 2012b,c). However, one of many significant questions remaining to be answered is whether or not neurons are capable of emulating levels of quantum computational performance to optimize the fitness of cellular decisions during both normal and challenged cytophysiological states. Experimental and theoretical findings concerning aneural ciliate heuristic-guided social behaviors tantalizingly imply that all eukaryotic cells equipped with cellular machinery for fast autocatalytic intracellular Ca^2+^ signaling and response regulation may execute quantum-efficient algorithms to select and implement appropriate response strategies to better cope with changing ambient and homeostatic conditions (Clark, 2010a,b,c,d, 2011, 2012b, 2013a). To partly address this issue for neurons, I argue in the present Hypothesis and Theory article that intracellular store-operated Ca^2+^ release offers a suitable and common mechanism for widespread biological evolution and expression of Grover's quantum algorithm in cellular life. I begin with brief reviews of intracellular store-operated Ca^2+^ release in neurons and the basic facets of Grover's quantum algorithm. I then narrow my discussion to highlight correspondences between a mathematical fire-diffuse-fire model of intracellular store-operated Ca^2+^ release and Grover's quantum algorithm, followed by an unprecedented, if preliminary, parameterization of the fire-diffuse-fire model to fit Grover's quantum algorithm operating specifications. Lastly, I contemplate testable model predictions and the ecological and evolutionary impact that a cellular version of Grover's quantum algorithm may have for healthy and diseased neurons and the organisms to which they are invested.

## Intracellular Ca^2+^ dynamics and response regulation in neurons

**Figure 1:**
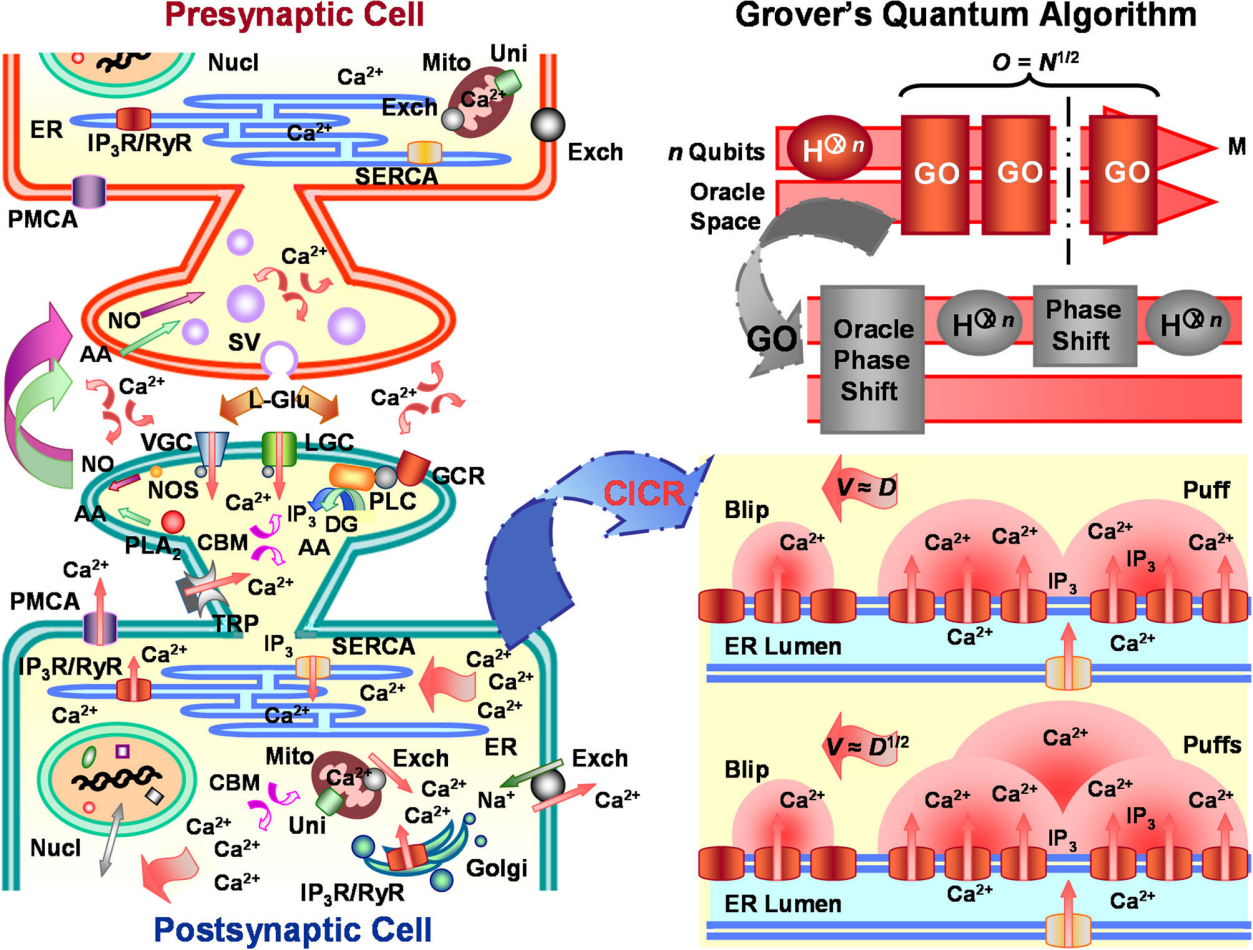
**Calcium-induced calcium reactions (CICRs) emulate Grover's quantum algorithm in neuronal information processing**. *Left panel* portrays major characteristic substrate (e.g., receptors, organelles, etc.) involved in Ca^2+^-mediated response regulation of arbitrary glutamatergic neurons, including, but not limited to, substrate critical for synaptic plasticity, cellular energetics, immunoprotection, homeostasis, gene expression, biosynthesis, molecular trafficking, cytoskeletal organization, and cell fate. Similar mechanisms affect both pre- and post-synaptic neurons, but, for descriptive purposes, post-synaptic cell activity is emphasized. Ca^2+^ entry into the post-synaptic neuron through voltage-gated receptor (VGC), ligand-gated receptor (LGC), and transient potential receptor (TRP) channels and stimulated inositol 1,4,5-trisphosphate (IP_3_) production by activated G-protein coupled receptors (GCR) help initiate cytosolic CICRs from integral IP_3_ receptors (IP_3_R) located along the endoplasmic reticulum (ER) membrane. CICRs may cause traveling waves of varying velocities and patterns which emulate search routines capable of eliciting/suppressing appropriate response regulation from different cellular compartments. *Lower right panel* illustrates CICR saltatory and continuous waves. Saltatory Ca^2+^ waves and the information they carry conduct at velocities (*V*) proportional to the classical Ca^2+^ diffusion coefficient (*D*). Whereas, faster continuous Ca^2+^ waves and the information they transmit move at velocities proportional to the square-root of the classical Ca^2+^ diffusion coefficient. Coefficient *D* of continuous waves for either intercluster or intracluster diffusion is assumed to be up to orders of magnitude greater than that for saltatory waves. The quadratic disparity in the velocities of saltatory and continuous waves corresponds to the root-rate increase of information processing by Grover's quantum algorithm over classical algorithms. *Upper right panel* shows schematic of Grover's quantum algorithm. The algorithm takes as input *n* qubits, upon which it performs Hadamard transformations (H^⊗*n*^) and Grover's operation (GO) to find a target *m* of *M* solutions stored in database *N*. Regardless of whether one or more consultations of the Oracle are needed, Grover's quantum algorithm finds the target solution within *O* = *N*^1/2^ algorithmic steps or operations *O*. Additional abbreviations: arachidonic acid (AA), Ca^2+^ binding molecule (CBM), Ca^2+^ uniporter (Uni), diacylgycerol (DG), Golgi apparatus (Golgi), L-glutamate (L-Glu), nucleus (Nucl), mitochondria (Mito), nitric oxide (NO), nitric oxide synthase (NOS), phospholipase A_2_ (PLA_2_), phospholipase C (PLC), plasma-membrane Ca^2+^ ATPase (PMCA), ryanodine receptor (RyR), sarcoplasmic-endoplasmic-reticulum Ca^2+^ ATPase (SERCA), Na^+^/Ca^2+^ exchanger (Exch), synaptic vesicle (SV).

Extensive varieties of functional Ca^2+^ channels, transporters, and exchangers are expressed by eukaryotic cells. Each protein type can be directly or indirectly involved in cellular response-regulatory pathways and/or Ca^2+^ homeostasis. The main classes of Ca^2+^ channels, transporters, and exchangers across animal phylogeny rely on mechanosensitive [e.g., transient receptor potential (TRP) compression and stretch receptors], ATP-dependent [e.g., sarcoplasmic-endoplasmic-reticulum (SERCA) and plasma-membrane ATPase (PMCA) Ca^2+^ uptake/extrusion pumps), ion-gated (e.g., Ca^2+^/H^+^ and Na^+^/Ca^2+^ exchangers)], voltage-gated (e.g., L-, N-, P/Q-, R-, and T-type receptors), ligand-gated [e.g., inositol 1,4,5-trisphosphate (IP_3_), ryanodine (Ry), and N-methyl-D-aspartate receptors (NMDA)], and peptidergic porin (e.g., aquaporins) mechanisms (Clark, 2013b; Clark and Eisenstein, 2013; Clark et al., 2013; Dolphin, 2006; Foskett et al., 2007). The majority of known selective or nonselective Ca^2+^ channel, transporter, and exchanger systems are well identified and studied for a range of differentiated animal cell types, such as neurons and myocytes. With respect to neurons (Figure 1, left panel), scientific attention is frequently given to those Ca^2+^-permeable or -activating proteins, such as L- and N-type voltage-gated channels, ligand-gated NMDA receptors (NMDARs) and α-amino-3-hydroxy-5-methyl-4-isoxazolepropionicacid receptors (AMPARs), and metabotropic glutamate receptors (GPCRs), critical for the induction and/or maintenance of certain forms of synaptic structural and transmission plasticity (cf. Franks and Sejnowski, 2002; Bear, 2003; Malenka and Bear, 2004; Iino, 2006; Cohen and Greenberg, 2008; Levitan, 2008; Yashiro and Philpot, 2008; Okamoto et al., 2009; Catterall, 2010; Selvaraj et al., 2010; Castillo et al., 2011; Fioravante and Regehr, 2011; Hartmann et al., 2011; Wright and Vissel1, 2012). However, these same and additional proteins are also instrumental, for example, in experience-independent cytokine and chemokine immunological responses, gene expression, cellular trafficking, and homeostasis (cf. Clark, 2013b; Clark and Eisenstein, 2013; Foskett et al., 2007), when extracellular Ca^2+^ influx and/or triggered IP_3_-dependent store-operated intracellular Ca^2+^-induced Ca^2+^ reactions (CICRs) help control endosome transport, membrane remodeling, and up- and downregulation of metabolic and catabolic processes. In classic scenarios of facilitated and depressed glutamatergic synaptic function, respectively known as long-term potentiation (LTP) and depression (LTD), extracellular Ca^2+^ enters the post-synaptic cell through activated NMDARs during the induction phase of plasticity. Ca^2+^ loading in dendritic spines often evokes either LTP or LTD in a concentration-dependent manner, with higher and lower levels of Ca^2+^ tending to respectively produce LTP and LTD (cf. Malenka and Bear, 2004). As free Ca^2+^ diffuses in spines and perhaps other cell compartments, various Ca^2+^-dependent messenger systems become activated and contribute to the induction and maintenance phases of plasticity. Signal transduction by Ca^2+^ and its sensors/binding proteins, such as calmodulin and calcinurins, stimulate cascading enzymatic activity from calcium-calmodulin kinase II (CaMKII) and IV, nitric oxide synthase, protein kinase C, tyrosine kinase Src, mitogen-activated protein kinase, and other molecular complexes that lead to enduring changes through post-synaptic CREB-dependent transcription and immediate early gene activation, post-synaptic receptor synthesis, transport, and distribution, pre- and post-synapse geometry, and pre-synaptic vesicular transport and docking at transmitter release zones (cf. Malenka and Bear, 2004). Since the number and spatial distribution of Ca^2+^-dependent LTP and LTD events can quickly exhaust NMDAR transients, LTP and LTD must be supported by store-operated CICRs (cf. Malenka and Bear, 2004; Verkhratsky, 2005). The initial requirements of Ca^2+^ loading to excite CICRs for expression of LTP and LTD differ according to the frequency of post-synaptic stimulation, whether LTP or LTD develops, and the type of neuron in which they occur. However, it is now accepted that IP_3_ receptor (IP_3_R)-mediated CICRs assist in directing response regulation under physiological constraints of neuronal synaptic transmission and plasticity.

## IP_3_Rs and cytophysiology of CICRs in neurons and other differentiated animal cells

Four integral 310-kDA tetrameric IP_3_R isoforms, all permeable to certain mono- and divalent cations, especially Ca^2+^, are expressed by animals (cf. Taylor et al., 2004; Foskett et al., 2007; Taylor and Tovey, 2010). Three of these isoforms, types 1 through 3, are encoded in vertebrate genomes (Patel et al., 1999; Taylor et al., 1999). Invertebrate genomes encode the remaining receptor isoform closely related to IP_3_R type 1 (Iwasaki et al., 2002; Ionescu et al., 2006). Except for structurally and functionally similar polymorphic ryanodine receptors (RyRs), IP_3_Rs are the foremost ion pore responsible for nonmitochondrial store-operated Ca^2+^ release in animal cells, including neurons. Receptors concentrate in the membrane of the ER (e.g., Ross et al., 1989; Otsu et al., 1990; Ferreri-Jacobia et al., 2005), the principle calcium storage site of most cells, but are also located in the nuclear envelope, Golgi apparatus, secretory vesicles, and plasma membrane of various differentiated cell types (Ross et al., 1989; Tanimura et al., 2000; Dellis et al., 2006). In mammalian brain, where IP_3_Rs are very rich in the cerebellum (Supattapone et al., 1988; Furuichi et al., 1989; Sharp et al., 1999) and hippocampus (Furuichi et al., 1993, 1994; Sharp et al., 1993), greater heterogeneity in intracellular distribution occurs for receptor isoforms. IP_3_R type 1, the most abundant isoform in brain, resides in dendrites, cell bodies, axons, and synaptic terminals of cerebellar Purkinje cells, while being largely confined to soma and proximal dendrites in other neurons (Ryugo et al., 1995; Dent et al., 1996; Sharp et al., 1999). The IP_3_R type 3, in contrast, is localized to neuropil and neuronal terminals (Sharp et al., 1999). Consistent with receptor distributions, IP_3_Rs act as a prominent signal interface between the ER and most other organelles, including mitochondria, to directly and indirectly affect cell processes (cf. Ponce-Dawson et al., 1999; Strier et al., 2003; Coombes et al., 2004; Taylor et al., 2004; Fraiman et al., 2006; Foskett et al., 2007; Solovey and Ponce-Dawson, 2010; Taylor and Tovey, 2010). Importantly, large observed variations in receptor structural identity may predictably correspond to an equally large functional diversity with subtle developmental and physiologic consequences for specific IP_3_R-populated organs and tissues. However, receptor types likely provide complementary and redundant substrate for intracellular Ca^2+^ signaling. Redundancy may be apparent in small observed divergences in agonist binding affinities across receptor types (cf. Foskett et al., 2007; Clark et al., 2013). Without significant variance in agonist sensitivity, separate isoforms are unable to titrate Ca^2+^-dependent physiological responses to differential cytosolic ligand concentrations. Moreover, channel phosphorylation and protein interactions tend to cause similar respective allosteric modification of heterotrophic ligand binding regardless of receptor type (cf. Foskett et al., 2007; Clark et al., 2013).

Well studied for oocyte development, myocardial activity, and cerebellar cortex neural output, IP_3_R-mediated ER Ca^2+^ release aids, for example, in regulating protein and lipid biosynthesis, cell energetics, stress responses, cell fate and death, synaptic plasticity, and immunoprotection (cf. Clark and Eisenstein, 2013). The complex spatiotemporal information conveyed in Ca^2+^ signals is highly dependent upon IP_3_ production by phospholipase C (PLC)-β and -γ, distribution and activation/inhibition properties of IP_3_Rs, uptake and storage of Ca^2+^ in the ER and other reservoirs, and influences over Ca^2+^ diffusion (cf. Clark and Eisenstein, 2013). The ER membrane encompasses a cisternal space that occupies about 10% of cell volume and importantly harbors cotranslational proteins, lipids, and divalent ions, such as Ca^2+^. ER-membrane-bound Ca^2+^ ATPases (e.g., sarcoplasmic-endoplasmic-reticulum Ca^2+^ ATPase) sequester free cytosolic Ca^2+^ in the ER lumen, where it stays free or becomes attached to buffers. Estimates of total luminal Ca^2+^ concentrations are as high as 1 mM. The fraction of unbound luminal Ca^2+^ ranges from 100 to 700 μM (Montero et al., 1995; Bygrave and Benedetti, 1996; Pinton et al., 1998; Alvarez and Montero, 2002; Bassik et al., 2004; Palmer et al., 2004; Verkhratsky, 2005). Although ER Ca^2+^ modulates IP_3_R function from the luminal side via interactions with channel sensor domains, cobinding of IP_3_ and Ca^2+^ to cytosolic sites opens the gated IP_3_R channel in a concentration-dependent manner, driving passive Ca^2+^ flux down its electrochemical gradient and into the cytosol. In IP_3_ concentrations ranging between 100 nM and 1 μM that continuously saturate receptor binding sites for IP_3_, Ca^2+^ generally excites and blocks IP_3_R activity in respective low (e.g., ~50 nM–1 μM) and high (e.g., >10 μM) concentrations (cf. Foskett et al., 2007). Changes in IP_3_R conformation and pore permeability occur due to IP_3_ and Ca^2+^ allosteric interactions that dissociate suppressor, calmodulin, and gatekeeper receptor regions, repositioning the transmembrane gate and activating Ca^2+^ conductance (cf. Foskett et al., 2007; Clark and Eisenstein, 2013). In absence of IP_3_ binding, low-affinity binding of cytosolic Ca^2+^ to one of two calmodulin heads occludes the ion channel as calmodulin crosslinks with suppressor and gatekeeper regions of adjacent receptor subunits. The receptor lumen stays closed and inactive when only cytosolic IP_3_ binds to receptor sites. Depending on recording preparations, receptor type, and other factors, Ca^2+^ conductance and current through single channels have been electrophysiologically measured at around 10–125 pS and 0.1–0.5 pA, respectively, (cf. Foskett et al., 2007). Maximum mean duration of IP_3_R opening tends to be no more than 15 and 40 ms for respective vertebrate and invertebrate receptor types (cf. Foskett et al., 2007). The lengths of these periods are independent of agonist concentrations. But the durations of subsequent prolonged refractory or reversible inactivation periods are determined by agonist concentrations. Frequency of channel activity and graded Ca^2+^ mobilization are thus primarily due to cytoplasmic ligand levels, with additional regulation by nucleotides, phosphorylation, redox states, and protein interactions.

Stimulation of an individual or several IP_3_Rs evokes a spatially discrete Ca^2+^ release usually termed a blip (Figure 1, lower right panel). Blips are the most elemental Ca^2+^ release event (cf. Foskett et al., 2007; Solovey and Ponce-Dawson, 2010). Quantal release creates a microdomain of high cytosolic Ca^2+^ concentration that can exceed 100 μM near the opening of an activated channel (Naraghi and Neher, 1997; Rios and Stern, 1997; Neher, 1998). Once a 10-μM Ca^2+^ threshold is reached, free cytosolic Ca^2+^ begins to exert inhibitory feedback control over all-or-none openings of surrounding IP_3_Rs. Inhibitory control of IP_3_Rs is proportionally tuned by presence of local IP_3_ concentrations, which interfere with the ability of Ca^2+^ to bind to low-affinity sites. Furthermore, rapid buffering by both mobile and immobile Ca^2+^ traps often limit diffusion of free cytosolic Ca^2+^ to a radius no greater than 5 μm from the source channel (Allbritton et al., 1992). Buffers alone are typically insufficient to quench a Ca^2+^ release event involving multiple adjacent receptors. But beyond the distance of 5 μm, cytosolic Ca^2+^ concentrations, homeostatically regulated by transporters, exchangers, and porins, steeply decline from 1 μM to ~50 nM without widespread Ca^2+^ mobilization (Naraghi and Neher, 1997; Rios and Stern, 1997; Neher, 1998). Since IP_3_Rs organize in autocatalyst-linked clusters on the ER membrane, a larger coordinated Ca^2+^ release event, commonly called a puff or spark, can be initiated following a blip (cf. Ponce-Dawson et al., 1999; Strier et al., 2003; Coombes et al., 2004; Fraiman et al., 2006; Foskett et al., 2007; Solovey and Ponce-Dawson, 2010). Puffs occur as Ca^2+^ from the blip diffuses and binds to neighboring inactive IP_3_Rs already docked by IP_3_ (Figure 1, lower right panel), an autocatalytic event referred to as a CICR or fire-diffuse-fire reaction (cf. Yao et al., 1995; Ponce-Dawson et al., 1999; Strier et al., 2003; Coombes et al., 2004; Taylor et al., 2004; Fraiman et al., 2006; Guisoni and de Oliveira, 2006; Shuai et al., 2006; Foskett et al., 2007; Bruno et al., 2009; Smith and Parker, 2009; Ur-Rahman et al., 2009; Solovey and Ponce-Dawson, 2010; Taylor and Tovey, 2010). Single IP_3_Rs are typically separated by 10–20 nm within a cluster of perhaps 50 receptors covering a maximum ER-membrane surface area of 400 nm^2^ in some cells (Shuai et al., 2006; Bruno et al., 2009; Ur-Rahman et al., 2009). Individual clusters can be separated by regular or irregular distances of up to around 2 μm (Yao et al., 1995). Though cytosolic Ca^2+^ binding proteins may alter Ca^2+^ transport in the vicinity of a receptor channel cluster, the interchannel distances are too short to prevent most ions from diffusing. Therefore, when an estimated 20 to 35 IP_3_Rs become simultaneously bound with coligands, Ca^2+^ puffs arise (Shuai et al., 2006; Bruno et al., 2009; Smith and Parker, 2009). As mentioned earlier, this autocatalytic coupling of clustered receptors forms local and global cellular networks or lattices that can generate either small- or large-scale mobilization of Ca^2+^. Irregularities in spatial organization of IP_3_R clusters together with channel coupling associated with CICRs produce assorted intracellular Ca^2+^ signals. The amplitude, frequency, and velocity of signals vary according to cytosolic Ca^2+^ buffer concentrations, feedforward excitation of IP_3_Rs via possible high affinity Ca^2+^ binding, feedback inhibition of IP_3_Rs via possible low affinity Ca^2+^ binding, and crosstalk with additional messenger systems (cf. Clark, 2011, 2012b), such as cAMP pathways (e.g., Siso-Nadal et al., 2009). Puffs may trigger global Ca^2+^ waves as cellular conditions promote sustained CICRs. Waves can oscillate, extinguish, and travel throughout different cell compartments as saltatory, continuous, or anisotropic fronts. The spatiotemporal variability of blips, puffs, and large-scale waves suggests a high degree of specificity is achieved for intracellular Ca^2+^ signaling, reducing the likelihood of corruption and loss of transmitted information content by noisy intracellular processes (cf. Clark, 2011, 2012b, 2013a). In many respects then, the behavior of neuronal CICRs conforms to fundamental principles and attributes of (classical and quantum) search algorithms and patterns used to efficiently find and execute various kinds of appropriate cellular responses to extracellular and/or intracellular stimuli (Clark, 2010a,b,c,d, 2011, 2012a,b, 2013a; Clark and Eisenstein, 2013).

## Description of Grover's quantum algorithm

Before entering into discussion on the relationship between CICRs and Grover's quantum algorithm, I now identify basic specifications of Grover's quantum algorithm through a short primer of information and computational theory. In standard quantum information theory and computation, the classical bit originated by Shannon (1948a,b) is replaced with the quantum bit or qubit, a concept and term, respectively credited to Weisner (1983) and Schumacher (1995). Qubits are information units that may be transmitted, transformed, stored, and measured. The possible states of a single qubit in Dirac notation are the orthonornal unit vectors or basis (eigen)states $|0\rangle =\left[\begin{array}{c} 1\\ 0 \end{array}\right]$ and $|1\rangle =\left[\begin{array}{c} 0\\ 1 \end{array}\right]$ which span a two dimensional vector, state, or Hilbert space. States |0〉 and |1〉 correspond to classical bit states of 0 and 1. However, unlike classical bits, these states may form an indefinite linear combination or superposition: |ψ〉 = *a*|0〉 + *b*|1〉, where variables *a* and *b* are complex numbers called vector amplitudes (cf. Nielsen and Chuang, 2000). By way of a common example useful to later exposition of Grover's quantum algorithm, states |0〉 and |1〉 forming the unique quantum supposition (|0〉 − |1〉)/2^1/2^ have respective amplitudes 1/2^1/2^ and −1/2^1/2^. If qubit states remain linearly independent, then any measurement or eigenvalue *m* on the system |ψ_*i*_〉 via arbitrary Hermitian measurement operators *M*_*i*_ ≡ |ψ_*i*_〉 〈ψ_*i*_| and *M*_0_ = *I* − ∑_*i* ≠ 0_ |ψ_*i*_〉 〈ψ_*i*_|, where *I* is the identity matrix $\left[\begin{array}{c} 1\\ 0 \end{array}\begin{array}{c} 0\\ 1 \end{array}\right]$, will decompose into a single pure state |0〉 or |1〉 with respective probabilities |*a*|^2^ and |*b*|^2^, so that measurement probability *p*_*i*_ = 〈ψ_*i*_|*M*_*i*_|ψ_*i*_〉 = |*a*|^2^ + |*b*|^2^ = 1 (cf. Nielsen and Chuang, 2000). Conversely, in agreement with the entropic uncertainty principle, an informational analog to Heisenberg's uncertainty principle, indistinguishable or nonorthonormal quantum states cannot be measured with certitude because of variances inherent in observables. As one may expect, these concepts become slightly more complicated in instances of composite or joint systems—those involving two or more qubits. Although the full implications of composite systems go beyond the scope of this article, it is important to introduce some content on the subject for future consideration. Take two entangled component systems, each respectively described by superposition states |ψ_*i*_〉 = (|0〉 + |1〉)/2^1/2^ and |ψ_*j*_〉 = (|0〉 − |1〉)/2^1/2^. The state space of such a bipartite system is defined by the tensor product |ψ_*i*_〉 ⊗ |ψ_*j*_〉 = |ψ〉_10_ = (|00〉 − |11〉)/2^1/2^, yielding, in the present case, the third Bell basis state or Einstein–Podolsky–Rosen pair. Composite quantum information systems, such as the four Bell basis states or Einstein–Podolsky–Rosen pairs, play significant roles in superdense coding, information encryption, error diagnosis and correction, and other aspects of quantum computation, including execution of quantum algorithms (cf. Nielsen and Chuang, 2000).

As previously noted, quantum computational methods entail use of specialized quantum gates and circuits to form algorithms that manipulate qubits to purposefully arrive at some goal state, much as would be accomplished for classical bits operated on by classical logic gates and circuits. Purposes may include, for instance, finding correct solutions to difficult or classically intractable factorization, ordering, counting, and search problems. Now imagine a quintessential large database or map of salient landmarks, such as cities (or, as will be detailed below, rate-limiting parameters for selective spatiotemporal chemical diffusion patterns). Using a classical search algorithm to discover the shortest route *n* among all possible routes *N* through every city on the map, a dilemma known as the Hamiltonian cycle decision problem, requires *N* total operations *O* or algorithmic steps [i.e., *O*(*N*)]. The same search problem may be accelerated to *O*(*N*^1/2^) with Grover's quantum algorithm and its unique Grover's operator (Grover, 1996) (Figure 1, upper right panel). Typical uses of Grover's quantum algorithm search register entries *i* = {*i*_*n*_|*n* = 0, …, *N* − 1} indexed to actual elements *n* of *N*. This convention is created so that the database can be conveniently set to *N* = 2^*n*^ bits of storage, with a subset *M* = 1 ≤ *M* ≤ *N* = {*i*_*m*_|*m* = 1 ≤ *m* ≤ *N* − 1} of exact solutions. Another convention enlists a function θ that accepts as input an integer *x* = *i* valued over the range 0 to *N* − 1. The function returns results θ(*x*) = 1 or θ (*x*) = 0 when a solution *m* is or is not obtained, respectively. Given these constraints, Grover's operator consists of four distinct sequential procedures (cf. Nielsen and Chuang, 2000): (1) application of the Oracle, (2) application of the Hadamard transformation, (3) application of a conditional phase shift, and (4) application of the Hadamard transformation. The operator first samples inputs from the initial equally weighted superposition state $|\psi \rangle =1/N^{1/2}\sum_{x=0}^{N-1}|x\rangle$ generated by the efficient Hadamard transform, H^⊗*n*^, then it labels problem solutions through the unitary action of the Oracle:

(1) $$|x\rangle (|0\rangle -|1\rangle )/2^{1/2}\overset{O}{\to }-1^{\theta (x)}|x\rangle (|0\rangle -|1\rangle )/2^{1/2},$$

where |*x*〉 represents the index-register qubit set to |0〉 and (|0〉 − |1〉)/2^1/2^ is the single Oracle qubit which assists in flipping or phase-shifting |*x*〉 only when θ(*x*) returns 1 as a result. A second Hadamard transformation is utilized following the Oracle call to place labeled qubits into superposition. From this state, a conditional phase shift, |*x*〉 → − (− 1)^θ(*x*)^|*x*〉, becomes executed for all basis states not equal to |0〉. The final Hadamard transformation again puts the register qubit into superposition for possible further Oracle summons in the event a target solution is not located, although successful search attempts may require no more than one Oracle call.

## Correspondence between CICRs and Grover's quantum algorithm

If modifiable operation of linked intracellular Ca^2+^ release sites and associated affector/effector systems function as quantum-like computational networks for response regulation, as reported by Clark (2010a,b,c,d, 2011, 2012a,b, 2013a), then reaction-diffusion equations should reveal classical and quantum properties of search algorithms and of search patterns selectively applied to those networks. One such reaction-diffusion equation, the simple fire-diffuse-fire model of Ca^2+^ propagation, defines Ca^2+^ waves by the following evolution equation (Ponce-Dawson et al., 1999):

(2) $$\begin{array}{l} \partial \left[{\text{Ca}}^{\text{2}+}\right]/\partial t=D\text{​}\left(\partial^{2}\left[{\text{Ca}}^{\text{2}+}\right]/\partial^{2}x\right)+\left(\sigma /d^{2}\tau \right)\\ \;\sum \delta \text{​}\left(x-x_{i}\right)H\text{​}\left(t-t_{i}\right)H\text{​}\left(t_{i}+\tau -t\right), \end{array}$$

where [Ca^2+^](*x, t*) is the average concentration of calcium in directions perpendicular to the direction *x* of propagation, δ(ζ) is the δ function, *H*(ζ) is the Heavyside step function (and not the Hadamard transform), *D* is the classical Ca^2+^ diffusion coefficient, *d* is the (mean) distance between Ca^2+^ release sites, *t*_*i*_ is the first time the *i*th Ca^2+^ release site reaches threshold value, σ is the total number of Ca^2+^ ions released per storage site, and τ is the period that receptors remain open to release Ca^2+^ in a single event. Notably, the evolution equation and parameters for this sort of model are general enough to fit conditions stipulating IP_3_R clusters or individual IP_3_Rs as Ca^2+^ release sites (cf. Guisoni and de Oliveira, 2006; Solovey and Ponce-Dawson, 2010), with corresponding changes in intersite distances, timescales, and released Ca^2+^ concentrations. The dynamics of the equation additionally rely on two dimensionless parameters, Γ and β (Ponce-Dawson et al., 1999), with:

(3) $$\Gamma =\left(\sigma /d^{3}\right)/\left({\left[{\text{Ca}}^{\text{2}+}\right]}_{T}-{\left[{\text{Ca}}^{\text{2}+}\right]}_{b}\right),$$

where σ/*d*^3^ is the concentration of Ca^2+^ released and [Ca^2+^]_*T*_ and [Ca^2+^]_*b*_ are respective threshold and basal concentrations of Ca^2+^, and

(4) $$\beta =\left(D/\tau \right)/d^{2}.$$

Parameter Γ of Equation 3 acts as a multiplicative variable governing the ease of starting a Ca^2+^ wave, the velocity at which it will travel, and, therefore, the effectiveness of the wave to effect response regulation. Whereas, the value of parameter β of Equation 4 defines whether a Ca^2+^ wave propagates with a slow saltatory, fast continuous, or intermediate mixed front. As chemical processes, such as the duration of Ca^2+^ mobilization or degree of Ca^2+^ buffer overload, become rate-limiting over interstore distances, β ≫ 1 and Ca^2+^ waves transition from saltatory to continuous waves (Figure 1, lower right panel). Moreover, saltatory wave propagation travels at a rate proportional to the Ca^2+^ diffusion coefficient, *v* ≈ (*D/d*)*g*^−1^Γ, where *g*^−1^ is an inverse function (Ponce-Dawson et al., 1999). In contrast, the velocity of continuous waves is proportional to the square-root of the Ca^2+^ diffusion coefficient, *v* ≈ (*D*/τ)^1/2^*f*^−1^Γ, where *f*^−1^ is an inverse function (Ponce-Dawson et al., 1999). When continuous waves have sufficiently large Γ, wave velocity approximates the Luther equation, *v* = α(*D*/τ)^1/2^ with α = Γ^1/2^. The equations for wave velocity should not be misleading, as continuous waves are often faster than saltatory ones (cf. Izu et al., 2001). (Noted exceptions include the large-scale completely homogenous fertilization waves of oocytes.) If the value of *D* is the same for both saltatory and continuous waves and τ is much larger for continuous waves, then saltatory Ca^2+^ waves would always transmit at faster speeds. But when compared to saltatory waves, continuous waves well exceed buffering capacities of slow and fast Ca^2+^-buffer species and display far greater diffusion coefficients and shorter intersite diffusion times (cf. Strier et al., 2003) for both intercluster and intracluster models of diffusion for constant small *d*. The effect of overcoming buffering capacity on continuous wave velocity may be also amplified by diminution of *τ* on the order of one to two magnitudes to the ms timescale (cf. Izu et al., 2001; Strier et al., 2003; Foskett et al., 2007), which differs from τ given by Ponce-Dawson et al. (1999). In either situation of saltatory or continuous waves, wave conduction generally fails for small *D* (e.g., <10 μm^2^/s), large *d* (e.g., *d* > 3 μm), and extremely small or large Γ (cf. Keizer et al., 1998; Ponce-Dawson et al., 1999; Strier et al., 2003). These values reflect significant differences in the physiological roles of saltatory and continuous Ca^2+^ waves (e.g., Keizer et al., 1998), with the former believed to inhibit local and global cellular responses via Ca^2+^-wave conduction failure and CICR blockade and the latter believed to evoke and integrate a range of local and global cellular responses through complex spatiotemporal patterns and widespread delivery of information throughout the cell. Fire-diffuse-fire models of Ca^2+^ propagation are remarkably robust, explaining the diffusive characteristics of store-operated Ca^2+^ regulation in a generous variety of eukaryotic cell types, including oocytes, cardiac myocytes, and neurons (e.g., Ponce-Dawson et al., 1999; Strier et al., 2003; Coombes et al., 2004; Timofeva and Coombes, 2004; Fraiman et al., 2006; Guisoni and de Oliveira, 2006; Thul et al., 2007; Solovey and Ponce-Dawson, 2010; Bressloff, 2014).

Importantly, the leading edge of intracellular Ca^2+^ gradients passing between separate receptor clusters is relatively slow, being experimentally recorded to typically travel at velocities ranging from 20 to 70 μm/s (Jaffe, 1993). Some reports, however, indicate continuous longitudinal waves can approach an astonishing 6000 μm/s in live cells (Miura et al., 1999). In any event, when *N* and *D* are numerically related (Clark, 2010a, 2012b), the quadratic disparity between the speeds (i.e., intersite-travel time) of saltatory and continuous Ca^2+^ waves appears consistent with expression of a square-root quantum algorithm that increases target searches, such as searching for the most appropriate response to external and/or internal stimuli, in living cells beyond the bounds of classical algorithms (Clark, 2010a,b, 2011, 2012b) (Figure 1, lower and upper right panels). In the algorithmic (rather than strictly physicochemical) sense, the properties of classical Ca^2+^ diffusion effectively describe a phenomenological basis for Grover's quantum algorithm (cf. Clark, 2010a,b). A physicochemical manifestation of quantum mechanics via a quantum diffusion term is unnecessary to produce quantum-efficient algorithm searches, a result perhaps counterintuitive for most biophysicists since diffusive processes may be classical, quantum, or semiclassical/semiquantum in nature. At biologically relevant subsecond times, warm temperatures, and micrometer scales described for the conditions of Ca^2+^ fire-diffuse-fire reactions (cf., Ponce-Dawson et al., 1999), quantum diffusive processes produce minor effects without thermodynamic shielding, such as in the case of bacterial photosynthetic reaction cores (Hu et al., 1998; Sener et al., 2005), or pump-process energy transfer, such as in the possible case of actomyosin polymerization (Matsuno, 1999). Neither thermodynamic shielding nor pump-process constraints must occur for initiation and maintenance of classical fire-diffuse-fire reactions. The diffusion coefficient or diffusivity, *D*, in Equations 2 and 4 is a purely classical parameter generally derived from Fick's laws and the Einstein-Smoluchowski relation as *D* = μ*k*_*B*_*T*, where μ is particle mobility or the inverse drag coefficient, *k*_B_ is Boltzmann's constant, and *T* is temperature in degrees Kelvin (cf. Clark, 2012b). This equation, via the Einstein-Sutherland relation, becomes the Einstein-Stokes equation in one dimension, *D* = *k*_B_*T*/2πη*r*, and in three dimensions, *D* = *k*_B_*T*/6πη*r*, for spherical particles of radius *r* moving through a fluid of viscosity η at a low Reynolds number. If the density of the diffusing material affects *D*, then the diffusion equation is nonlinear and *D* is taken to be variable (cf. Clark, 2012b). When independent of thermodynamic influences, *D* = *h*/4π*m*, where *h* is Planck's constant and *m* is the mass of the diffusing particle. The latter definition of the diffusion coefficient is quantum mechanical (cf. Clark, 2012b). One may draw direct comparison of these sorts of effects with the operation of closely related technological quantum networks performing search functions (e.g., Bianconi and Barabási, 2001; Bianconi, 2002a,b, 2003; Stella et al., 2005; Clark, 2010b,c,d, 2011, 2012a,b, 2013a). In such instances, observed statistical quantum-like outcomes, often referred to as quantum mechanical analogs, emerge from weighted macroscale computational networks and their parameters capable of both classical and quantum behavior. For technological systems (Bennett, 2003; Ladyman et al., 2007) and individual cells (Clark, 2010b,c,d, 2011, 2012b, 2013a; Bérut et al., 2012; Mehta and Schwab, 2012), this behavior is consistent with Landauer's principle of energy/information transfer. Similar to computational network analogs of quantum behavior, the classical Ca^2+^ diffusion coefficient might instantiate a computational analog of quantum mechanical systems without actually residing in a physicochemical quantum regime.

Although the physical expression of the diffusion coefficient should be entertained, it *must be stressed* that in some sense any reflection is superfluous with respect to application of Grover's quantum algorithm. The reason for this, as indicated in the previous paragraph, is that a relationship between *N* and *D* seems apparent (and will be established in below sections). The variable *N* represents the total number of search elements queried by Grover's quantum algorithm. Its value is neither quantum nor classical! Therefore, *D* needs to be neither quantum nor classical to effect Grover's quantum algorithm in a cellular system! That is, what makes Grover's quantum algorithm quantum in nature is its action on a search field, not necessarily the properties of the search field itself. In view that classical diffusion terms satisfy the quadratic improvements needed for Grover's quantum algorithm, it is interesting that search selectivity by such an algorithm in single cells may be enhanced, instead of being damped, by diffusion barriers sometimes causing unstable Ca^2+^ gradients. Anisotropic patterns of Ca^2+^ diffusion due to free cytosolic chaperons and buffers (Chen et al., 2008, 2009) and frequency and amplitude modulated Ca^2+^ liberation (De Pitta et al., 2008, 2009) have been reported to help improve the specificity of encoding sensory information transmitted by intracellular Ca^2+^ cascades. Intracellular spaces are filled with Ca^2+^ traps, such as immobile binding sites. Though traps reduce the effective diffusion coefficient below expected values for free diffusion, propagation of information can move faster than single particle diffusion (Pando et al., 2006). The storage and retrieval of that information is expected to be further refined by recursive phosphorelays affecting Ca^2+^ permeability to extracellular sources and subsequent reactivation CICRs (Clark, 2010a,b,c,d, 2011, 2012a,b, 2013a). Collectively, these findings should be put into local and global contexts of mixed and continuous wave fronts rather than taken to mean that spatiotemporal patterns of ineffectual punctuate store-operated Ca^2+^ emissions or unreliable saltatory Ca^2+^ waves serve as useful media to convey information vital to response regulation (cf. Keizer et al., 1998). These events likely rather function as wave guides that direct mixed or continuous waves to specific target locations within cell compartments.

## Parameterizing the neuronal fire-diffuse-fire model for Grover's quantum algorithm

The superficial similarity between variables *N* and *D* in quadratic processing efficiency encourages mathematical treatment that supplants analogy and parameterizes the Ca^2+^ fire-diffuse-fire model to more precisely fit specifications of Grover's quantum algorithm (Clark, 2011, 2012b). Because the model captures local dynamics of individual IP_3_R permeability, which inherently control the evolution of collective wave behavior within and across networked IP_3_R clusters, Grover's quantum algorithm may be reduced to the scale of a single receptor channel, as opposed to receptor clusters or an entire ER membrane of receptors. This convention has several attractive qualities. First, an inability to make quantum measurements due to quick decoherence rates of superposed states becomes a negligible confound for quantum logic operators the physical size of tetrameric protein channels (cf. Beck and Eccles, 1992; Gutin et al., 1996; Turin, 1996; Cieplak and Hoang, 2003; Davies, 2004; Brookes et al., 2007; Solov'yov et al., 2012). Second, uncovering (or framing) a reasonable search goal and associated parameters becomes more conceptually manageable. In regard to both issues, the natural inclination would be to perhaps equate *N* from the previous example of the Hamiltonian cycle decision problem to the total number of possible spatiotemporal patterns of chemical diffusion needed to evoke a proper IP_3_R-mediated neuronal response to external and/or internal perturbation, with the target solution being the shortest chain or route of networked receptor clusters across the entire or a circumscribed area of the ER membrane surface. While this decision-problem situation correctly assumes that some or all receptor clusters can be activated as a collective search routine to evoke an arbitrary desired cell response, it challenges the spatiotemporal limits of superposed (physical and not network-analog) quantum states needed to execute Grover's quantum algorithm, since each state embodies a long-range networked pattern of catalyst-linked receptor clusters (e.g., minimally greater than 2 or 4 μm) and, consequently, probably surpasses Wigner's mass-time uncertainty for periodic mechanical processes (cf. Wigner, 1957, 1981; Reimers et al., 2009). The mechanism and processing efficiency of this kind of emergent search algorithm likely would be characteristically (physicochemically) classical in nature. Whereas, the almost instantaneous and continuous chemical diffusion in an approximate 10- to 20-nm distance between two activation-primed IP_3_Rs (Shuai et al., 2006; Bruno et al., 2009; Smith and Parker, 2009) avoids violating Wigner-type quantum boundaries (cf. Pešić, 1993; Schwartz et al., 2005) and yields suitable conditions for expression of a quantum algorithm. Moreover, in terms of second messenger reaction-diffusion cascades and response regulation, stochastic punctate intracluster spatial patterns of diffusion often play significant roles in spark and wave initiation and are complemented by intermediate and overloaded ion-concentration magnitudes, which help force activation gradients, fast conduction velocities, and stable propagation to initiate cell responses (cf. Keizer et al., 1998; Izu et al., 2001; Strier et al., 2003; Chen et al., 2008, 2009; Solovey and Ponce-Dawson, 2010). Stipulating individual IP_3_Rs, and their quantum-mechanical small reaction sites and gating kinetics (cf. Ahern et al., 2009; Chan et al., 2010; Pitt et al., 2010; Li et al., 2013), as the computational apparatus of Grover's quantum algorithm allows one then to resolve the search problem to finding the shortest time or fastest rate *m* taken to reach and autocatalytically activate a neighboring receptor from among a database *N* of all possible time and activation outcomes (Table 1). Given slower intracluster saltatory Ca^2+^ waves have high propagation and response-regulation failure rates (e.g., Guisoni and de Oliveira, 2006; Solovey and Ponce-Dawson, 2010), target solutions will be elements of the set *M* of fast intracluster continuous Ca^2+^ waves. Such a query is idealized by the search for the maximum Ca^2+^ diffusion coefficient *D*_max_, which, in integer form spanning the range of possible integer diffusion coefficients, bears likeness to *N*. However, as individual IP_3_Rs do not actually detect diffusion coefficients, the search must be conducted over a “register” indexing each different *D* with a concentration-dependent parameter biologically associated with *D* and germane to IP_3_R reaction kinetics.

**Table 1:** **Comparison of major Ca^2+^ fire-diffuse-fire model and Grover's quantum algorithm parameters**.

Ca^2+^ fire-diffuse-fire model^*^	**Grover's quantum algorithm**
**DIFFUSION**	
*D* = (β*d*^2^)/τ = μ*k*_B_*T*	
*D*_S_ = *vdg*/Γ = {*D*_*S*_*l*__|*l* = min, …, max}, with *D*_*S*_min__ > 0, *D*_*S*_max__ ≪ *D*_*C*_min__	
*D*_C_ = τ(*vf*/Γ)^2^ = {*D*_*C*_*l*__|*l* = min, …, max}, with *D*_*C*_min__ ≫ 0, *D*_*S*_max__ and *D*_*C*_max__ ≪ ∞	*D*_*C*_ = (*D*_*S*_max__ ≪ *M* ≤ *N*) = {*i*_*m*_|*m* = *D*_*S*_max__ ≪ *m* = *N* − 1}
*D*_max_ = 0 ≤ *D*_max_ ≤ *D*_*C*_max__ = {*D*_*n*_*j*__|*j* = 0, …, *D*_*C*_max__}	*D*_max_ → *N* = {*n*_*j*_|*j* = 0, …, *D*_*C*_max__}, one-to-one discrete nearest integer mapping
**WAVE DYNAMICS**	
β = (*D*/τ)/*d*^2^	
β_*S*_ = (*D*_*S*_/τ)/*d*^2^	
β_*C*_ = (*D*_*C*_/τ)/*d*^2^	
β_max_ = (*D*_max_/τ)/*d*^2^	
Γ = (σ/*d*^3^)/([Ca^2+^]_*T*_ − [Ca^2+^]_*b*_)	
Γ_*S*_ = τ *vg*/β*d* = {Γ_*S*_*l*__|*l* = min, …, max}, with Γ_*S*_min__ > 0, Γ_*S*_max__ ≪ Γ_*C*_min__	Γ_*S*_ = *i*_*F*_ = {*if|f* = 0 ≤ *f* < Γ_*C*_min__}
Γ_*C*_ = *vf*/β^1/2^*d* = {Γ_*C*_*l*__|*l* = min, …, max}, with Γ_*C*_min__ ≫ 0, Γ_*S*_max__ and Γ_*C*_max__ ≪ ∞	Γ_C_ = *i*_*M*_ = {*i*_*m*_|*m* = Γ_*C*_min__ ≤ *m* ≤ Γ_*C*_max__}
Γ_max_ = {*i*_*n*_|*n* = 0, …, Γ_*C*_max__}	Γ_max_ = *i* = {*i*_*n*_|*n* = 0, …, *N* − 1}

* *Model variables are suitable for describing both local (interreceptor or intracluster) and global (intercluster) intracellular waves*.

To make the present exposition more explicit and amenable with the previous coverage of Grover's quantum algorithm, I now mathematically define critical variables *N, M*, and *D* (Table 1), giving fuller attention to physical descriptions of the Grover's quantum algorithm workspaces and operators in subsequent paragraphs (Figure 1, upper right panel). As before, *N* = {*n*_*j*_|*j* = 0, …, ∞}, but, as will be determined below, is realistically a finite interval with respect to reaction-diffusion parameters. Elements *n*_*j*_ of *N* are indexed with register entries *i* = {*i*_*n*|*n* = 0, …, *N* − 1_}, with a subset *M* = 1 ≤ *M* ≤ *N* = {*i*_*m*_|*m* = 1 ≤ *m* ≤ *N* − 1} of exact solutions to the search problem. Algebraic manipulation of Equation 4 yields the general definition of the classical Ca^2+^ diffusion coefficient:

(5) $$D=\left(\beta d^{2}\right)/\tau .$$

However, fluctuations in loading of cytosolic Ca^2+^ buffering systems and, more appreciably, choice of rate-limiting parameters (inter-IP_3_R) *d*^2^, τ, and σ for the fire-diffuse-fire model make the diffusion coefficient variable. Sharper definitions of *D* may be obtained from the wave velocity equations for saltatory Ca^2+^ waves, *v* = (*D/d*)*g*^−1^Γ, and continuous Ca^2+^ waves, *v* = (*D*/τ)^1/2^*f*^−1^Γ. It follows that the respective diffusion coefficients for saltatory and continuous Ca^2+^ waves are:

(6) $$D_{\text{S}}=vdg/\Gamma =\{D_{S_{l}}|l=\min,\ldots ,\max\},$$

with *D*_*S*_min__ > 0 and *D*_*S*_max__ ≪ *D*_*C*_min__, and

(7) $$D_{\text{C}}=\tau {\left(vf/\Gamma \right)}^{2}=\{D_{C_{l}}|l=\min,\ldots ,\max\},$$

with *D*_*C*_min__ ≫ 0, *D*_*S*_max__ and *D*_*C*_max__ ≪ ∞. Setting *D*_max_ = 0 ≤ *D*_max_ ≤ *D*_*C*_max__ = {*D*_*n*_*j*__|*j* = 0, …, *D*_*C*_max__} and considering the one-to-one discrete mapping *D*_max_ onto *N, D*_max_ → *N, M* therefore transforms into the subset *D*_*C*_ = *D*_*S*_max__ ≪ *M* ≤ *N* = {*i*_*m*_|*m* = *D*_*S*_max__ ≪ *m* ≤ *N* − 1} of all correct solutions involving only intracluster continuous Ca^2+^ wave fronts. A *major* result from this interpretation is that, in order to arrive at a solution *m* = *D*_*C*_, quadratic and exponential speed-ups in respective algorithmic search time and wave velocity must coexist, with a maximum algorithmic search time of *O*(*D*_max_/*D*_*C*_)^1/2^ and wave velocity of *v*_C_ = *v*^2^_*S*_! In addition, Equations 6 and 7 are especially useful for purposes of implementing Grover's quantum algorithm, since they are inversely proportional to dimensionless parameter Γ rather than β. As shown in Equation 3, reliance upon Γ permits Ca^2+^ diffusion coefficients to be indexed to physiologically pertinent ratios involving Ca^2+^ concentrations liberated by IP_3_R-dependent store operation and free cytosolic Ca^2+^ concentrations sensed by multiaffinity IP_3_R cytosolic Ca^2+^ binding sites. So that, index values of register entries *i* corresponding to *D*_max_ and *D*_*C*_ may be practically redefined by substituting Equation 5 into Equations 6 and 7, then solving for Γ for separate saltatory and continuous Ca^2+^ wave forms:

(8) $$\Gamma_{\text{S}}=\tau vg/\beta d=\{\Gamma_{S_{l}}|l=\min,\ldots ,\max\},$$

with Γ_*S*_min__ > 0 and Γ_*S*_max__ ≪ Γ_*C*_min__, and

(9) $$\Gamma_{\text{C}}=vf/\beta^{1/2}d=\{\Gamma_{C_{l}}|l=\min,\ldots ,\max\},$$

with Γ_*C*_min__ ≫ 0, Γ_*S*_max__ and Γ_*C*_max__ ≪ ∞. Therefore, index *i* = {*i*_*n*_|*n* = 0, …, *N* − 1} = Γ_max_ = {*i*_*n*_|*n* = 0, …, Γ_*C*_max__} and solution subset index *i*_*M*_ = {*i*_*m*_|*m* = Γ_*C*_min__ ≤ *m* ≤ Γ_*C*_max__}. Remember also from earlier reviewed content that Grover's quantum algorithm employs a function θ that accepts as input an integer *x* = *i* valued over the range 0 to *N* − 1. The function returns results θ(*x*) = 1 or θ(*x*) = 0 when a solution *m* is or is not acquired, respectively. In parameterizing the fire-diffuse-fire model for Grover's quantum algorithm, elements of the index set *i* yielding θ(*x*) = 1 readily signify the solution subset *i*_*M*_ = {*i*_*m*_|*m* = Γ_*C*_min__ ≤ *m* ≤ Γ_*C*_max__} for intracluster continuous Ca^2+^ waves, whereas elements of the index set *i* yielding θ(*x*) = 0 signify the incorrect-solution or failure subset *i*_*F*_ = {*i*_*f*_|*f* = 0 ≤ *f* < Γ_*C*_min__} for all intracluster noncontinuous Ca^2+^ waves, including saltatory and possibly mixed Ca^2+^ wave fronts.

**Figure 2:**
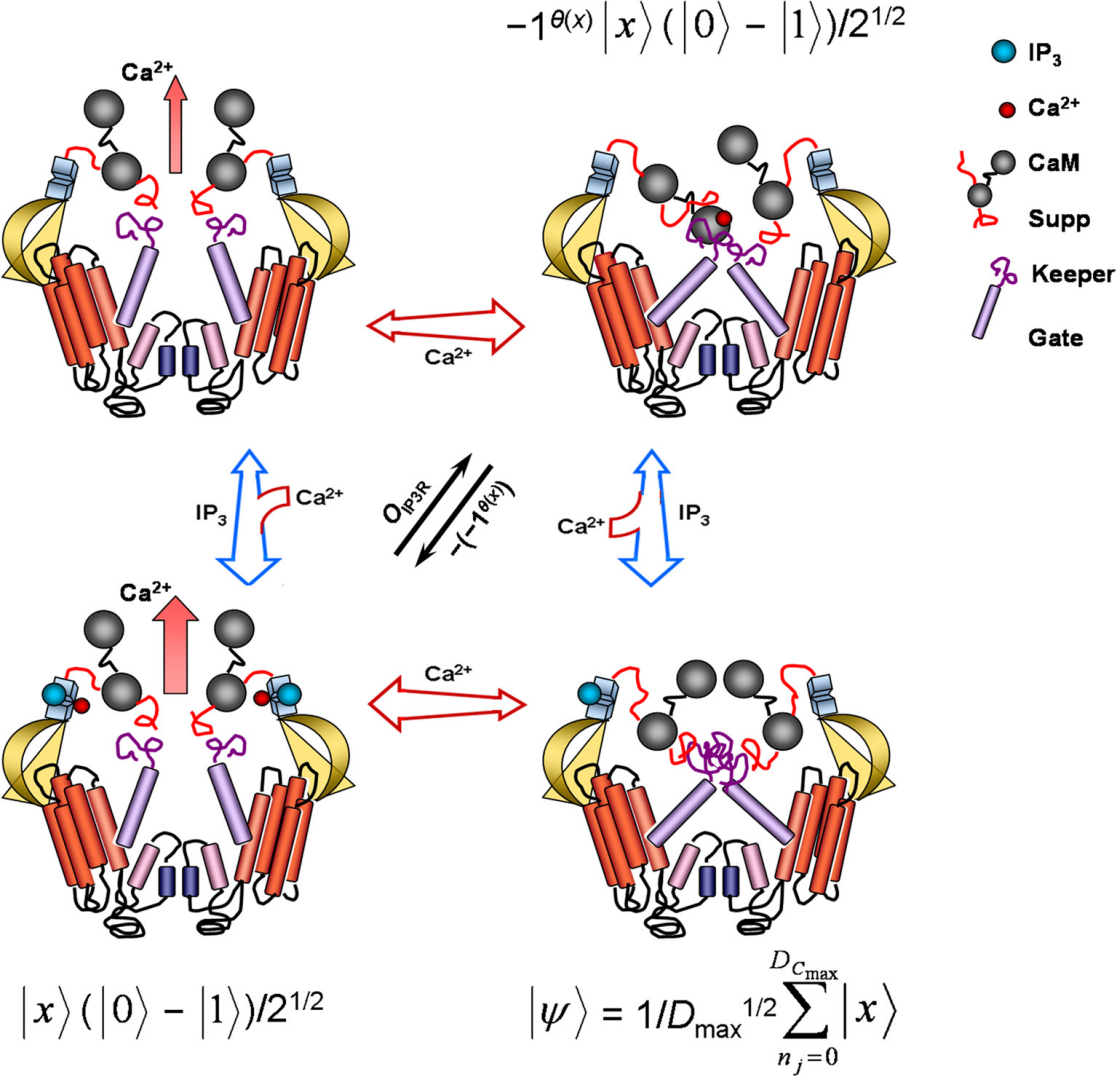
**Model of conformation, ion permeability, and corresponding Grover's quantum-algorithm function of an inositol 1,4,5-trisphosphate receptor channel (IP_3_R)**. Each cross-section contains two of four complete IP_3_R subunits. When only cytosolic IP_3_ (blue sphere) binds, the receptor lumen stays closed and inactive. Cobinding of cytosolic IP_3_ and Ca^2+^ (red sphere) to separate high-affinity sites proximal to the IP_3_-binding domain dissociates suppressor (Suppr), calmodulin (CaM), and gatekeeper (Keeper) regions, repositioning the transmembrane gate (Gate) and activating Ca^2+^ conductance. In absence of IP_3_ binding, low-affinity binding of cytosolic Ca^2+^ to one of two calmodulin heads occludes the ion channel as calmodulin crosslinks with suppressor and gatekeeper regions of adjacent receptor subunit. No ligand binding is accompanied by a small leaking Ca^2+^ conductance. Free cytosolic proteins, nucleotides, and other substances can facilitate or impair IP_3_R gating by interacting with the IP_3_ binding-core, suppressor, and gate-keeper regions. Free endoplasmic-reticulum proteins and Ca^2+^ may also further modulate pore activity (not shown) via selectivity filters (small blue cylinders) located near pore helices (small rose cylinders). In the superposition state |ψ〉, the IP_3_R samples all possible index values Γ_max_ marking *D*_max_, returning an output *x* denoting a successful or unsuccessful search for shortest times or fastest rates *m* needed for store-released concentrations of free cytosolic Ca^2+^ to continuously diffuse and autocatalytically activate a nearest neighbor receptor. This superposition state may be regarded indefinitely stable in saturating IP_3_ concentrations. A phase shift by *O*_IP3R_ reversibly inactivates the receptor channel with high free cytosolic Ca^2+^ concentrations. Another subsequent phase shift reactivates the channel, confirming solution *m* has been found. See Equation 10 and relevant text for additional details. IP_3_R conformation representations adapted from Clark and Eisenstein (2013) with permission.

With key fire-diffuse-fire model parameters written in terms of Grover's quantum algorithm, candidate physicochemical substrate for algorithm operators can be next identified to a first approximation using known IP_3_R molecular biology and function (Figure 2). Grover's quantum algorithm, as previously mentioned, requires five distinct sequential procedures. The first of these steps, in notation consistent with the fire-diffuse-fire model, is application of the Hadamard transformation, H^⊗*n*_*j*_^, which prepares the algorithm in an initial equally weighted superposition state $|\psi \rangle =1/D_{\max}{}^{1/2}\sum_{n_{j}\;=\;0}^{D_{C_{\max}}-1}|x\rangle$. Such a state presumes that a single IP_3_R acting as Grover's quantum algorithm is capable of simultaneously detecting any probable index values Γ_max_ = {*i*_*n*_} marking *D*_max_ = {*D*_*n*_*j*__} and associated with *x*, the variable denoting a successful or unsuccessful search for shortest times or fastest rates *m* needed for store-released concentrations of free cytosolic Ca^2+^ to continuously diffuse and autocatalytically activate a nearest neighbor receptor and, thereby, ensure fast cellular response regulation. Furthermore, IP_3_R molecular regions and all other substrate effecting the superposition interrogate the superposition state with interaction-free measurements to maintain the superposition state until a solution is determined (e.g., Hosten et al., 2006). This computational feat is, of course, accomplished via inferential measurement of *D*_max_ via measurement of index Γ_max_ inherent in the operation of IP_3_Rs. Although IP_3_Rs may switch between four different conformational states, the inactivated IP_3_-bound IP_3_R conformation is perfectly suited for the initial superposition state of Grover's quantum algorithm because all saltatory and continuous waves affiliated with Γ_max_ retain *equal* probabilities of manifesting (Figure 2).

After initializing the IP_3_R into this IP_3_-saturated superposition state, which may remain indefinitely so in saturating IP_3_ concentrations, the final four steps of Grover's quantum algorithm involve execution of Grover's operator—application of the Oracle, a second Hadamard transformation, a conditional phase shift, and the last Hadamard transformation. Grover's operator may be expected to sample |ψ〉 and to then mark problem solutions through the unitary action of the IP_3_R Oracle:

(10) $$|x\rangle (|0\rangle -|1\rangle )/2^{1/2}\overset{O_{IP_{3}R}}{\to }-1^{\theta (x)}|x\rangle (|0\rangle -|1\rangle )/2^{1/2},$$

where again |*x*〉 denotes the index-register qubit set to |0〉 (i.e., all *i*_*f*_ values, including value 0 for the inactivated IP_3_-bound IP_3_R conformation) and (|0〉 − |1〉)/2^1/2^ is the superposed IP_3_R-Oracle qubit. The IP_3_R Oracle phase-shifts |*x*〉 only when θ(*x*) returns 1 as a result. Function θ(*x*), accordingly, may be thought of as the low- and high-affinity Ca^2+^ binding sites located at the cytosolic end of the IP_3_R emulating Grover's quantum algorithm (Figure 2). Recall that IP_3_R activity demonstrates a bell-shaped response profile to cytoplasmic Ca^2+^ levels. Ca^2+^ generally excites and blocks IP_3_Rs in respective low (e.g., ~50 nM–1 μM) and high (e.g., >10 μM) concentrations. Low Ca^2+^ concentrations capable of inducing sustained Ca^2+^ waves bind to high-affinity binding sites, changing cation-pore permeability by repositioning the transmembrane gate and activating Ca^2+^ conductance. In contrast, very low or very high Ca^2+^ levels keep the IP_3_R in a closed state. Ca^2+^ binding sites operating as θ(*x*) return 1 upon ion-pore opening and otherwise return 0 as a result. Coincident with θ(*x*) action and all-or-none IP_3_R stimulation, the IP_3_R Oracle labels |*x*〉 by flipping the state from |1〉 to |0〉. Isolating the Oracle's operation to a particular IP_3_R molecular region and function presents some conceptual difficulties. Arguments could be made for cytoplasmic and ER-lumen channel sensors which detect the presence of cations ions and IP_3_R interactions with nucleotides, proteins, and other substrate. Regardless, as noted above, once an IP_3_R becomes active, respective high quantal Ca^2+^ release exceeding 10 μM near the opening of the same channel begins to cause reversible autoinhibition, presumably via low-affinity Ca^2+^ binding sites located proximal to IP_3_R cytoplasmic terminus. Inactivation may last longer than the open-channel period *τ*. Such an effect is tantamount to the |*x*〉 phase shift produced by the Oracle of Grover's quantum algorithm (Figure 2). Furthermore, channel inactivation spans the entire Γ_max_ index set, placing the IP_3_R into another superposition state |ψ〉, as expected from application of the second Hadamard transformation. When IP_3_R inactivation is finally reversed to a fully excited Ca^2+^-permeable state, the conditional phase shift, |*x*〉 → − (− 1)^θ(*x*)^|*x*〉, has been performed for all basis states not equal to |0〉. The last Hadamard transformation puts the register qubit into an equally weighted superposition for possible future Oracle summons. Since τ can be accurately determined to be greater than the time of intersite Ca^2+^ diffusion for continuous waves (Strier et al., 2003; Solovey and Ponce-Dawson, 2010) and since the IP_3_R-Oracle phase shift of |*x*〉 only serves to emphasize timescale differences implicit in Γ of saltatory and continuous Ca^2+^ waves, the IP_3_R-mediated fire-diffuse-fire model simulating Grover's quantum algorithm will find target *m* indexed to *i*_*m*_ with quadratic improvement in search efficiency. Together, steps 3 to 5 of Grover's quantum algorithm may be expressed as:

(11) $$H^{⊗n_{j}}(2|0\rangle \langle 0|-I)H^{⊗n_{j}}=2|0\rangle \langle 0|-I,$$

where *I* is the identity matrix (cf. Nielsen and Chuang, 2000). The overall effectiveness of arriving at a solution involving a fast intracluster continuous Ca^2+^ wave predictably grants neurons better opportunities to initiate local and global response regulation for a variety of necessities.

## Testable predictions and significance of Grover's quantum algorithm for other CICR models

A fundamental prediction of the above analytical treatment unaddressed by standard classical interpretations is that, for an individual IP_3_R-based Grover's quantum algorithm to arrive at a solution *m* = *D*_*C*_ upon sensing/actuating Γ_max_ with multiaffinity IP_3_R cytosolic Ca^2+^ binding sites and gating kinetics, quadratic and exponential speed-ups in respective algorithmic search time and wave velocity must coexist, with a maximum algorithmic search time of *O*(*D*_max_/*D*_*C*_)^1/2^ and wave velocity of *v*_*C*_ = *v*^2^_*S*_. If these constraints are not met, algorithm-processing capabilities will approach a classical algorithm taking *O*(*D*_max_/*D*_*S*_) time. After only quick inspection, the velocity equations for saltatory, *v*_*S*_ = (*D/d*)*g*^−1^Γ, and continuous waves, *v*_*C*_ = (*D*/τ)^1/2^*f*^−1^Γ, may falsely imply to readers that a quantum-search result is impossible. Indeed, the arguments of Ponce-Dawson et al. (1999), for instance, enforce the idea that (global or intercluster) stable saltatory wave propagation is the fastest mode of transmission, at least for oocyte maturation. That conclusion heavily relies on the condition of τ_*S*_ < τ_*C*_, where receptor-channel open duration τ_*C*_ might be one to two orders of magnitude larger than τ_*S*_. This type of variation in receptor open time is not experimentally reported for IP_3_Rs, which tend to be open for a fixed period between 15 and 40 ms depending on receptor subtype irrespective of Ca^2+^ wave propagation mode (Foskett et al., 2007; Clark and Eisenstein, 2013). And, if τ is instead calculated to be the rise time of Ca^2+^ concentration across a local grouping of channels contributing to a release event, the divergence between τ_*S*_ and τ_*C*_ can be expected to be no more than one magnitude for most neuronal Ca^2+^ waves. Taking these aspects into consideration and utilizing examples of experimentally realistic parameter values (e.g., Izu et al., 2001; Strier et al., 2003; Foskett et al., 2007; Clark and Eisenstein, 2013) for *d* = 2 μm between IP_3_R clusters, *D* = 190 μm^2^/s for continuous waves, *D* = 15 μm^2^/s for saltatory waves, and τ = 0.04 s for both continuous and saltatory waves, it becomes apparent continuous waves (β = 1.9, *v* ≈ (*D*/τ)^1/2^ ≈ 69 μm/s) can exceed saltatory wave (β = 0.15, *v* ≈ *D*/*d* ≈ 7.5 μm/s) velocity by greater than a power of 2. Despite being a simple example, these values underscore the plausibility of fast continuous Ca^2+^ waves displaying characteristics consistent with those predicted by the Grover's quantum-algorithm model. Importantly, the version of the fire-diffuse-fire model used in the present article assumes deterministic channel refractivity and instantaneous buffering for σ without specification of Ca^2+^ store re-uptake or extracellular extrusion (cf. Ponce-Dawson et al., 1999). Fast and slow Ca^2+^ buffering and Ca^2+^ extrusion and sequestration, such as that parameterized in stochastic models (e.g., Coombes and Timofeeva, 2003; Coombes et al., 2004; Keener, 2006), will decelerate and even quench wave propagation, especially lower concentration saltatory waves. But the impact buffering has on wave-conduction modality and velocity is further dependent on model selection. For instance, in the rapid-Ca^2+^-buffering approximation with or without slow re-uptake (e.g., Strier et al., 2003), the fire-diffuse-fire model further becomes susceptible to error when describing saltatory-wave evolution. Continuous waves, however, largely overcome buffering capacity, even more so when a massive wave-induction event occurs, such as calcium overload from extracellular sources or large ER mobilization. These sorts of effects predictably serve to increase Γ, effective diffusion, wave velocity, and thus fast stable Ca^2+^ wave search patterns at scales of receptor clusters or larger ER membrane surface areas supported by the Grover's quantum-algorithm model. In situations where waves are quenched or annihilated through Ca^2+^ buffers, re-uptake, or other factors (Keizer et al., 1998; Thul et al., 2007), the affected area will act as a wave guide to direct the (stochastic or deterministic) initiation and movements of subsequent waves, including planar, spiral, and oscillatory wave profiles capable of sending and storing distinct types of cellular information. With respect to these contexts (cf. Falcke, 2003a), future detailed numerical examination of parameters is needed to identify the continuum limits of fire-diffuse-fire saltatory and continuous wave dynamics and their relation to expression of Grover's quantum algorithm at different scales of CICR organization. As a special case of percolation Ca^2+^-release/-diffusion universality (cf. Timofeva and Coombes, 2004; Solovey and Ponce-Dawson, 2010), fire-diffuse-fire models conform to different spatiotemporal scales. The Grover's quantum algorithm model, in its conceptual form as a single IP_3_R operator, is best understood through local CICRs conducting at the scale of single receptor clusters, so that the model may reside in a quantum regime at both thermodynamic and informational degrees of freedom. For local or intracluster wave propagation mode and velocity, where *d* = 20 nm, the set of above (valid) values for global (i.e., intercluster) continuous and saltatory waves yield respective local (intracluster) continuous waves of β = 19000 with *v* ≈ 69 μm/s and β = 1500 with *v* ≈ 19 μm/s. The large values for β indicate conditions supporting saltatory waves at a global scale do not necessarily ensure presence of saltatory waves for intracluster dimensions. Thus, although the algorithm searches for and finds optimal solution *m*, the boundary between local and global waves needs to be better resolved through numerical examination of Γ_max_—the critical Grover's quantum-algorithm index of effective diffusion and solution *m*.

Though a computationally tractable term containing many essential traits, employing the deterministic threshold-dependent Γ_max_ unsatisfactorily weakens the explanatory power of the current model in regard to channel gating kinetics and quantum molecular action underlying formation and interrogation of superposition states, such as |ψ〉. Resolution of state boundaries for the IP_3_R Grover's quantum-algorithm model therefore should be perfected by redefining Γ_max_ and, naturally, constituent Γ to include buffering, re-uptake, and gating kinetics terms, as is done with more complex wave-evolution equations (e.g., Strier et al., 2003; Timofeva and Coombes, 2004; Thul et al., 2007). Deterministic (DeYoung and Keizer, 1992) and probabilistic (Falcke, 2003b) mathematical models of single IP_3_R behavior offer richer accounts of the dynamic range expected for channel activity, wave profiles, feedback control, and search patterns inherent in different channel reaction kinetics and structural configurations (Figure 2). Accordingly, Equation 3 may be restated, for benefit of future investigations, as:

(12) $$\Gamma =\left(\Phi_{1}-\Phi_{2}-\Phi_{3}\right)/\left([{\text{Ca}}^{2+}]\omega_{t_{i}}-[{\text{Ca}}^{2+}]\omega_{t_{0}}\right).$$

Variables Φ_1_, Φ_2_, and Φ_3_ quantify changes in point-source store-operated Ca^2+^ emission through respective release, re-uptake, and buffering kinetics relative to threshold activation kinetics, ([Ca^2+^]ω_*t*_*i*__ − [Ca^2+^]ω_*t*_0__), with [Ca^2+^]ω_*t*_*i*__, [Ca^2+^]ω_*t*_0__ = [Ca^2+^]_*T*_ − [Ca^2+^]_*b*_. The role of molecular dynamics in channel state is evident in each Φ (cf. DeYoung and Keizer, 1992; Falcke, 2003b); Φ_1_ = *C*_ER/C_(*F*_MOpO_ + *F*_*L*_)([Ca^2+^]_ER_ − [Ca^2+^]_C_), Φ_2_ = *F*_U_[Ca^2+^]^2^_*C*_/[Ca^2+^]^2^_*C*_ + *A*^2^, and Φ_3_ = *K*_B_[Ca^2+^]_C_[B]_C_, where *C*_*ER/C*_ is the ratio of Ca^2+^ in ER and cytoplasmic volumes, *F*_MO_ is maximum outward IP_3_R Ca^2+^ flux, *F*_L_ is IP_3_R Ca^2+^ leak flux, *F*_U_ is maximum ER Ca^2+^ uptake, *p*_O_ is the probability of IP_3_R-channel opening, *A* is the ER-uptake-activation constant, *K*_B_ is the Ca^2+^-buffer-binding constant, [Ca^2+^]_ER_ and [Ca^2+^]_CF_ are free ER and cytoplasmic Ca^2+^ concentrations, and [B]_C_ is the free Ca^2+^ buffer concentration. While the complexity of these equations may be increased, addition of the open-channel-probability term,

(13) $$\begin{array}{l} p_{\text{O}}=[\left([{\text{Ca}}^{2+}]\left[{\text{IP}}_{3}\right]K_{Ca_{I}}\right)/(\left[{\text{Ca}}^{\text{2}+}\right]\left[{\text{IP}}_{3}\right]+\left[{\text{IP}}_{3}\right]K_{Ca_{I}}\\ \;+{K_{IP3_{1}}K_{Ca_{I}}+\left[{\text{Ca}}^{\text{2}+}\right]K_{IP3_{2}})\left(\left[{\text{Ca}}^{\text{2}+}\right]+K_{Ca_{A}}\right)]}^{3}, \end{array}$$

where *K*_*Ca*_*I*__ is the dissociation constant for IP_3_R channel inhibition, *K*_*IP3*_1__ and *K*_*IP3*_2__ are IP_3_ dissociation constants, and *K*_*Ca*_*A*__ is the dissociation constant for IP_3_R channel activation, gives a much stronger framework for molecular considerations. For example, a consequence of the new Γ formulation is that the initialized index-register qubit becomes $|x\rangle =1/4^{1/2}\sum_{Mn\;=\;0}^{4-1}|100\rangle$. Vector |100〉 is the molecular configuration of composite IP_3_R binding subunits for each IP_3_R monomer (*Mn*) primed for channel activation and Ca^2+^ conductance. The first, second, and third vector columns of |100〉 represent the respective (saturated) occupied IP_3_-biding subunit (i.e., 1), the unoccupied high-affinity Ca^2+^-binding subunit (i.e., 0), and the unoccupied low-affinity Ca^2+^-binding subunit (i.e., 0). When multiqubit-controlled function θ(*x*) = 1 = |110〉, the high-affinity subunit binds Ca^2+^, making the IP_3_R permeable to cations. The IP_3_R Grover's operator, also acting as a multiqubit operator through coligand channel gating kinetics, as earlier conjectured, then labels |*x*〉 by flipping the final two or all column values in conjunction with saturating- or subsaturating-IP_3_, Ca^2+^-dependent channel inactivation. The operators's rate and probability of flipping between 0 and 1 for each column and of finding target solution *m* is expectedly proportional to percent saturation of [Ca^2+^]_*C*_ for high- and low-affinity Ca^2+^-binding subunits and, therefore, proportional to index Γ_C_, *D*_C_, and *v*_C_.

The IP_3_R-based Grover's quantum algorithm of CICR behavior and Ca^2+^-mediated cellular response regulation thus imparts greater comprehensiveness than possible with traditional single receptor, intracluster, and intercluster models. For instance, above quantum depictions of the IP_3_R conformational state vector |*x*〉 uniquely permits study of computationally efficient subcellular superdense coding (Clark, 2010c), quantum learning and memory (Clark, 2010a,b,c,d, 2011, 2012a,b, 2013a; Liu et al., 2013), quantum error diagnosis and correction (Clark, 2010c, 2013a), and quantum encryption (Clark, 2010c, 2013a, in press). Recalling the discussion on the third Bell state, (|00〉 − |11〉)/2^1/2^, the IP_3_R Grover's quantum-algorithm model may be extended to quantum coupling between two nearest neighbor IP_3_-saturated activation-primed receptor channels *R*_1_ and *R*_2_. For *R*_1_ and *R*_2_, each in superposition state $|x\rangle =1/4^{1/2}\sum_{Mn=0}^{4-1}|100\rangle$, the entangled bell bases may be deemed bidirectional coupling factors, such as Ca^2+^ sensitivity, shared between receptors and imposed by level of cytoplasmic modulator (e.g., ATP) saturation on coligand-dependent IP_3_R allostery (Foskett et al., 2007; Clark and Eisenstein, 2013), where *R*_1_ and *R*_2_ each possess one unique highly correlated complimentary Bell-state qubit. *R*_1_, depending on the action of multiqubit-operators θ and Grover's iteration, may send classical information intrinsic to Γ to *R*_2_ in the form of two-bit strings, such as 00 indicating neither sets of Ca^2+^ binding sites are occupied, 10 indicating only the set of high-affinity Ca^2+^ binding sites are occupied, or 01 indicating only the set of low-affinity Ca^2+^ binding sites are occupied. By employing the decoding gate matching *R*_1_'s classical signal, *R*_2_ can decode the superdense signal sent from *R*_1_ with less computational expenditure and superior acquired content than possible with classical information processing (cf. Nielsen and Chuang, 2000)^1^. Not only does this interpretation of channel conformational state and channel-channel communication agree with the IP_3_R Grover's quantum algorithm and CICR cytophysiology, it also establishes conditions for, among other phenomena, the expression of quantum-molecular memory storage and retrieval at levels of single IP_3_Rs and IP_3_R clusters. Since only three of four IP_3_R monomers must be activated for channel opening, the revised definition of |*x*〉 implies, in a manner like Ventura and Martinez (1999), single IP_3_Rs must maintain or remember *Mn*!/(*Mn* − 1)! = 4 superposed combinations of: (1) initial activation-primed conformational patterns |100〉 for about 19 ms or less to finish θ (*x*)-labeling before IP_3_ dissociates from its IP_3_R binding subunit, (2) θ(*x*)-transformed conformational patterns |110〉 for about 600 ms or less to finish the Oracle call before Ca^2+^ dissociates from its high-affinity IP_3_R binding subunits, and (3) Oracle-transformed conformational patterns |101〉 or |001〉 for about 5 s or less to finish the algorithm's final phase shift before Ca^2+^ dissociates from its low-affinity IP_3_R binding subunits (DeYoung and Keizer, 1992). These superposition states, embedded in |ψ〉, have spatiotemporal estimates well within quantum-decoherence bounds calculated for macromolecules located in live cells constrained by physiologically salient environments (cf. Gutin et al., 1996; McFadden and Al-Khalili, 1999; Cieplak and Hoang, 2003; Davies, 2004) and are experimentally testable by molecular dynamics and CICR simulation as well as bioassays involving wildtype and selectively mutated IP_3_R isoforms reconstituted in planar lipid bilayers. Although coverage of larger memory structures, such as that formed by multiply coupled IP_3_R intracluster patterns, is beyond the scope of this article, the same concepts presented for individual IP_3_Rs are scalable to intracluster dimensions and organization.

## Relevance of Grover's quantum algorithm for healthy and diseased neurons

In preceding sections, a fire-diffuse-fire model capable of explaining intracluster activity of individual IP_3_Rs was identified and analytically parameterized as a candidate mechanism for a natural neuronal form of Grover's quantum algorithm. Model accuracy fundamentally depends upon the sensitivity of IP_3_Rs to physiological parameters characterizing Ca^2+^-channel molecular structure and function as well as scalable quantum-level gains in classical Ca^2+^ diffusion rates, Ca^2+^ wave propagation, and appropriate fast cellular response regulation. Computations made by the IP_3_R algorithm infer search target solutions for fast classical Ca^2+^ diffusion rates via interrogation of index variable Γ_max_ associated with detectable free cytosolic Ca^2+^ concentrations and properties of IP_3_R channel conductance. Collectively, these features of the model allow the algorithm to operate within quantum computational and thermodynamic regimes without concern of incurring statistical mechanics measurement problems, such as decoherence of processed superposed eigenstates. The model nevertheless yields only a computational first-approximation of Grover's quantum algorithm and needs future refinement by applying sophisticated: (1) relativistic quantum physicochemistry theory to aptly match IP_3_R protein structure and function with the workspaces and operators of Grover's quantum algorithm, and (2) fire-diffuse-fire or lattice-percolation mathematical treatments of intracluster IP_3_R activity and CICR dynamics to fully address aspects of reaction-diffusion stochasticity and cytosolic Ca^2+^ buffering (cf. Izu et al., 2001; Strier et al., 2003; Guisoni and de Oliveira, 2006; Solovey and Ponce-Dawson, 2010). With respect to the latter topic, the present model becomes particularly relevant during actuation of intracellular compartmental Ca^2+^ loading from interstitial and intracellular cation sources. Even as a preliminary construct, the model implies contexts coincident with moderate to massive fluxes of Ca^2+^ through cation-permeable integral cell membrane pores and gated channels, such as during synaptic plasticity (Malenka and Bear, 2004), microbial pathogen attack (Clark, 2013b; Clark and Eisenstein, 2013; Clark et al., 2013), pathological oxidative stress (Bénédicte et al., 2012; Clark, 2012c), and neurological disease and aging (Verkhratsky, 2005; Bezprozvanny and Mattson, 2008; Stutzmann and Mattson, 2011), will assist in driving neurons to accelerate response regulation to quantum-level efficiency through induction of stable local and possibly subsequent global continuous Ca^2+^ waves. From a physiological perspective, dramatic increases in Ca^2+^ wave velocity and signal transduction at either intracluster or intercluster physical dimensions are impressive and attainable for a all sorts of differentiated eukaryotic cells (cf. Izu et al., 2001), requiring a maximum algorithmic search time of *O*(*D*_max_/*D*_C_)^1/2^ and wave velocity of *v*_*C*_ = *v*^2^_*S*_ to be realized. However, whether it is activated by stochastic blips of high Ca^2+^-conductance or deterministic cellular Ca^2+^ loading, a neuronal version of Grover's quantum algorithm, just like that proposed for other eukaryotic cells, figures to promote advantageous subcellular superdense coding (Clark, 2010c), quantum learning and memory (Clark, 2010a,b,c,d, 2011, 2012a,b, 2013a; Liu et al., 2013), quantum error diagnosis and correction (Clark, 2010c, 2013a), and quantum encryption (Clark, 2010c, 2013a, in press).

Equally significant, the quantum computational value of a receptor-scale Grover's quantum algorithm also can be expected to contribute to surprising classical information processing over much longer intracellular distances and times common to global, multicompartmental Ca^2+^ signaling. This expectation, unaccounted for by standard (stochastic or deterministic) CICR models, agrees with expression of three-agent quantum teleportation over communication channels transmitting classical bits via the information content of Γ, a circumstance, as described in the above section, that exposes superdense coding and quantum molecular memory for IP_3_R systems. Limited evidence suggests digital representation of cellular processing is encoded, transmitted, and stored by free intracellular Ca^2+^ (Plieth, 2005), CaMKII holoenzyme (Hameroff et al., 2010), and other Ca^2+^-related substrate broadly distributed across affector-effector systems. Individual molecules conveying bitwise information may form higher-order bytes at large concentrations and/or with molecular complexes, such as that reported for six-domain CaMKII encoding of microtubule lattices (Hameroff et al., 2010). Expression of an IP_3_R-based Grover's quantum algorithm, which may serve as a quantum amplifier and router, supports an estimated forty-fold boost in classical information processing by networked Ca^2+^ release sites through buffer-dependent superadditive Ca^2+^ wave densities and velocities (cf. Izu et al., 2001). Such effects presumably enhance operational traits of, for example, signal coincidence detection and integration, bidirectional synaptic plasticity, gene expression, immunodefenses, growth and tropisms, protein modification and transport, cytoskeletal polymerization, endosome formation and other cell functions by rapidly selecting, ordering, and/or counting optional local response regulation choices. The impact on neuronal cyctoskeleton operation alone attests to favorable cascading effects governing geometry of dendritic spines and synaptic cleft widths, intracellular molecular and vesicular trafficking, membrane repair, synaptogenesis, neurite growth, and efficacious synaptic transmission (e.g., Malenka and Bear, 2004; Verkhratsky, 2005; Bezprozvanny and Mattson, 2008; Craddock et al., 2010; Priel et al., 2010; Dent et al., 2011). Using archetypal glutamatergic neurons (e.g., Verkhratsky, 2005; Hagenston and Bading, 2011), which are exquisitely sensitive to fluctuations in intracellualar Ca^2+^ homeostasis, one can readily extrapolate how the IP_3_R-based Grover's quantum algorithm figures to help execute ER-dependent signal amplification and integration in healthy cell states of all neurons (Clark, 2012b). Unlike spontaneous punctate store-operated Ca^2+^ emissions observed for every eukaryotic cell or environmentally triggered massive store-operated Ca^2+^ overload observed for certain cell types (e.g., oocytes), large temporary elevation of Ca^2+^ microdomain concentrations following post-synaptic Ca^2+^ entry through activated NMDARs initiates widespread and differential response regulation in dendritic, somal, and axonal compartments. The amount and spread of inward Ca^2+^ current is too small to effect most transduction processes. Instead, NMDAR-mediated Ca^2+^ entry stimulates secondary Ca^2+^ release from intracellular stores. Information processed by NMDAR-dependent Ca^2+^ cascades is augmented by glutamate diffusion to extrasynaptic GPCRs responsible for intracellular IP_3_ generation. Compared to RyRs, IP_3_Rs tend to have greatest density in ER membranes located in the soma and dendritic shafts of neurons, where coligand IP_3_ may exert maximum influence (cf. Stutzmann and Mattson, 2011). Thus, an IP_3_R Grover's quantum algorithm likely guides heterosynaptic activity as well as kinase-induced (e.g., CaMKII and IV) gene transcription and protein synthesis accompanying longer-term structural plasticity, chiefly that of LTP (cf. Fitzjohn and Collingridge, 2002), rather than vesicular events particular to shorter-term presynaptic paired-pulse and post-tetanic facilitation. The algorithm ensures synaptic plasticity maintenance and nuclear response regulation by selecting the best target solutions for fast classical Ca^2+^ diffusion rates, stable continuous Ca^2+^ wave modes, and, consequently, activation of kinase and other messenger pathways via detectable free cytosolic Ca^2+^ concentrations and properties of IP_3_R channel conductance.

However, the appearance of a quantum-efficient search algorithm in the function of neurons or, for that matter, any other cell type need not necessarily guarantee evolutionary and/or ecological benefit for the cell that implements the algorithm and the host to which the cell belongs. Indeed, viral, bacterial, fungal, and protozoal infectious agents, including, among other pathogens, Human Immunodeficiency Virus type 1 and *Trypanosoma* parasites, that selfishly coopt neuron intracellular Ca^2+^ systems can use the algorithm to optimize timing and effectiveness of infection stages against barriers to invasion, pathogenesis, proliferation, and release. While most pathogens deploy well-timed Ca^2+^-dependent trophic and deleterious strategies, such as genomically encoded proteins and lipopolysacchrides, to exploit host-cell physiology, infected neurons are unusually susceptible to metabolic distress, apoptosis, and additional harmful effects leading to host cognitive impairments (Clark, 2013b; Clark and Eisenstein, 2013; Clark et al., 2013). Similarly, an uninfected, but diseased or aging, neuron can diminish its own cell performance by speeding-up the selection and execution of cellular response regulation incompatible with cell or host survival. Good examples involve neurodegenerative Parkinson's and Alzheimer's diseases, where ER-mediated deficits play major roles in disease severity and progression (cf. Stutzmann and Mattson, 2011). In the case of Alzheimer's disease, persistent upregulation of intracellular Ca^2+^ concentrations are known to commence and accelerate synaptic loss and amyloid plaque disposition, manifest pathocytological characteristics of the disease. While the excitotoxic nature of Ca^2+^ dysregulation in Alzheimer's disease is well understood, the exact causes are not (Stutzmann and Mattson, 2011; Popugaeva and Bezprozvanny, 2013). Recent and somewhat controversial evidence indicates that IP_3_R activation by presenilins, protein products of autosomal inherited mutated genes PS1 and PS2 linked to early onset Alzheimer's disease, may be a major contributing factor. The corresponding heightened IP_3_R-mediated Ca^2+^ mobilization means implementation of IP_3_R Grover's quantum algorithm drives diseased, injured, and/or aging neurons toward faster catastrophic failure than would be otherwise possible with classical response regulation. Hence, the delicate balance between beneficial and detrimental uses of a neuronal version of Grover's quantum algorithm demands thorough theoretical and empirical scrutiny of cellular conditions governing the algorithm's application in both healthy and diseased states.

### Conflict of interest statement

The author declares that the research was conducted in the absence of any commercial or financial relationships that could be construed as a potential conflict of interest.

## Footnotes

^1^In this example of Bell state |κ〉 10 = (|00〉 + |11〉)/2^1/2^, the first |00〉 and second |11〉 qubits respectively denote equilibrium and high Ca^2+^ sensitivity induced by saturating and supersaturating free cytoplasmic ATP. *R*_1_, which possesses the first qubit of coupling factor |κ〉_10_, applies θ(*x*) = 1 = |*x*〉 = |110〉, interacts |*x*〉 with its half of the coupling factor, and transmits classical bit-string 10 via Γ. *R*_2_, which possesses the second qubit of coupling factor |κ〉_10_, will next employ its half of the coupling factor and associated quantum-flip gate, θ(*x*) = 0 → 1 = |*x*〉 = |110〉, to recover information about the conformational state |*x*〉 of *R*_1_ and initiate its own Grover's operator. This computational scenario, resilient to error, is equivalent to three-agent quantum teleportation transpiring over classical communication channels (cf. Nielsen and Chuang, 2000).
